# Dedifferentiation maintains melanocyte stem cells in a dynamic niche

**DOI:** 10.1038/s41586-023-05960-6

**Published:** 2023-04-19

**Authors:** Qi Sun, Wendy Lee, Hai Hu, Tatsuya Ogawa, Sophie De Leon, Ioanna Katehis, Chae Ho Lim, Makoto Takeo, Michael Cammer, M. Mark Taketo, Denise L. Gay, Sarah E. Millar, Mayumi Ito

**Affiliations:** 1 The Ronald O. Perelman Department of Dermatology and Department of Cell Biology, New York University Grossman School of Medicine, New York, NY USA; 2 Division of Advanced Research Technologies, New York University Grossman School of Medicine, New York, NY USA; 3 Colon Cancer Program, Kyoto University Hospital–iACT, Kyoto University, Kyoto, Japan; 4 Black Family Stem Cell Institute, Department of Cell, Developmental and Regenerative Biology and Department of Dermatology, Icahn School of Medicine at Mount Sinai, New York, NY USA; 5 Present Address: DLGBioLogics, Paris, France

**Keywords:** Skin stem cells, Ageing

## Abstract

For unknow reasons, the melanocyte stem cell (McSC) system fails earlier than other adult stem cell populations^1^, which leads to hair greying in most humans and mice^2,3^. Current dogma states that McSCs are reserved in an undifferentiated state in the hair follicle niche, physically segregated from differentiated progeny that migrate away following cues of regenerative stimuli^4–8^. Here we show that most McSCs toggle between transit-amplifying and stem cell states for both self-renewal and generation of mature progeny, a mechanism fundamentally distinct from those of other self-renewing systems. Live imaging and single-cell RNA sequencing revealed that McSCs are mobile, translocating between hair follicle stem cell and transit-amplifying compartments where they reversibly enter distinct differentiation states governed by local microenvironmental cues (for example, WNT). Long-term lineage tracing demonstrated that the McSC system is maintained by reverted McSCs rather than by reserved stem cells inherently exempt from reversible changes. During ageing, there is accumulation of stranded McSCs that do not contribute to the regeneration of melanocyte progeny. These results identify a new model whereby dedifferentiation is integral to homeostatic stem cell maintenance and suggest that modulating McSC mobility may represent a new approach for the prevention of hair greying.

## Abstract (web summary)

Local microenvironmental cues modulate melanocyte stem cells, which control hair pigmentation, to enter different differentiation states, shifting between hair follicle stem cell and transit-amplifying compartments, a process that is different to other self-renewing systems.

## Main

Mammalian tissue regeneration largely depends on the capacity of adult stem cells to differentiate. Stem cell differentiation is generally viewed as unidirectional and follows the hierarchical model originally established through the study of haematopoietic stem cells^9–11^. This theory proposes that stem cells (undifferentiated state) have two distinct fates: one to sustain themselves through self-renewal and the second to produce transit-amplifying (TA) progeny (intermediate differentiated state) that ultimately give rise to functional differentiated cells during tissue regeneration^12–14^. In this model, the life-long durability of self-renewing tissues is typically sustained by a functionally and molecularly heterogeneous pool of stem and progenitor cells.

The organization of the McSC system, responsible for hair pigmentation, is thought to parallel that of hair follicle stem cells (HFSCs)^5–8^. McSCs are located in the bulge and hair germ (HG) area in telogen-phase hair follicles (HFs)^4,5^, where they are surrounded by HF epithelial stem cells (bulge cells)^14^ and progenitor cells (HG cells)^15,16^ that constitute to the McSC niche. At the onset of the anagen growth phase, McSCs regenerate differentiated melanocytes that migrate downwards into the hair bulb, where they produce pigment for the hair. Similar to HG epithelial cells, HG McSCs activate WNT signalling and undergo differentiation at the onset of regeneration^7^. Furthermore, McSCs in the bulge cycle more slowly than those in the HG during HF regeneration^6^. On the basis of these studies, McSCs in the bulge are postulated to represent long-term stem cells^6^. However, their distinct functions and self-renewal capacities have yet to be characterized. Despite the close relationship between HFSCs and McSCs, there are disparities in their durability over time: McSCs become exhausted earlier than HFSCs in most animals and humans, which results in hair greying during ageing^1–3^. The high prevalence of hair greying suggests that there may be specific disadvantages in the long-term maintenance of McSCs.

## HG McSCs can regenerate all melanocyte compartments

**Fig. 1:**
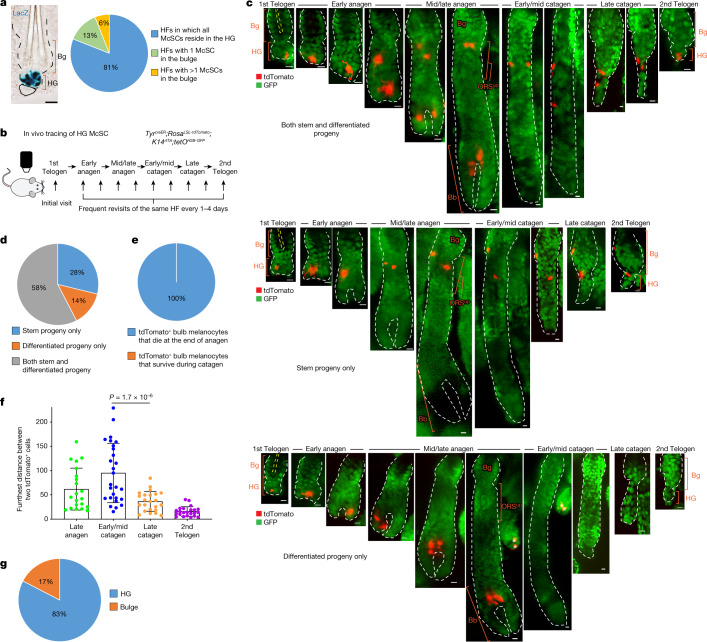
HG McSCs possess self-renewal ability. **a**, Left, bright-field image of a X-gal-stained HF from a *Dct*^*LacZ*^ mouse at telogen. Right, quantification of McSCs in specified locations in telogen HFs from *Dct*^*LacZ*^ mice. *N* = 52 single, whole HFs from 3 mice. **b**, Timeline of in vivo imaging of single HG McSCs in *Tyr*^*creER*^*;Rosa*^*LSL-tdTomato*^*;K14*^*rtTA*^*;tetO*^*H2B-GFP*^ mice. HFs containing single HG tdTomato-labelled cells were identified at the first telogen stage and then revisited at indicated stages. **c**, Live *z*-stack images of three representative examples of tdTomato^+^ HG McSCs that undergo distinct fates. K14^+^ epithelial cells (GFP^+^) are in green. See Extended Data Fig. 2 for additional examples. Yellow dashed line outlines club hair. White asterisk marks tdTomato^+^ cell in unrelated HF (bottom). **d**, Quantification of distinct fates of HG McSCs. *N* = 59 HFs from 7 mice. **e**, Percentages of tdTomato^+^ bulb MCs that die at the end of anagen or survive during the catagen phase. *N* = 20 HFs from 2 mice. **f**, Bar graph showing the furthest distance between two tdTomato^+^ cells in the bulge/ORS^up^ (at late anagen and early/mid catagen) and bulge/HG (at late catagen and second telogen). Data are presented as the mean ± s.d. *P* values (one-way analysis of variance (ANOVA) with Bonferroni multiple comparison test) are indicated, with 95% confidence interval at 35.81–81.13. *N* = 21 (late anagen), 27 (early/mid catagen) or 23 (late catagen and telogen) HFs from 2 mice. **g**, Percentages of tdTomato^+^ McSCs residing in bulge or HG at second telogen. *N* = 60 HFs from 6 mice. Dashed black or white lines outline the epithelial–dermal boundary (**a**,**c**). Scale bars, 20 μm (**a**) or 10 μm (**c**). Bb, bulb; Bg, bulge. Source data

**Extended Data Fig. 1:**
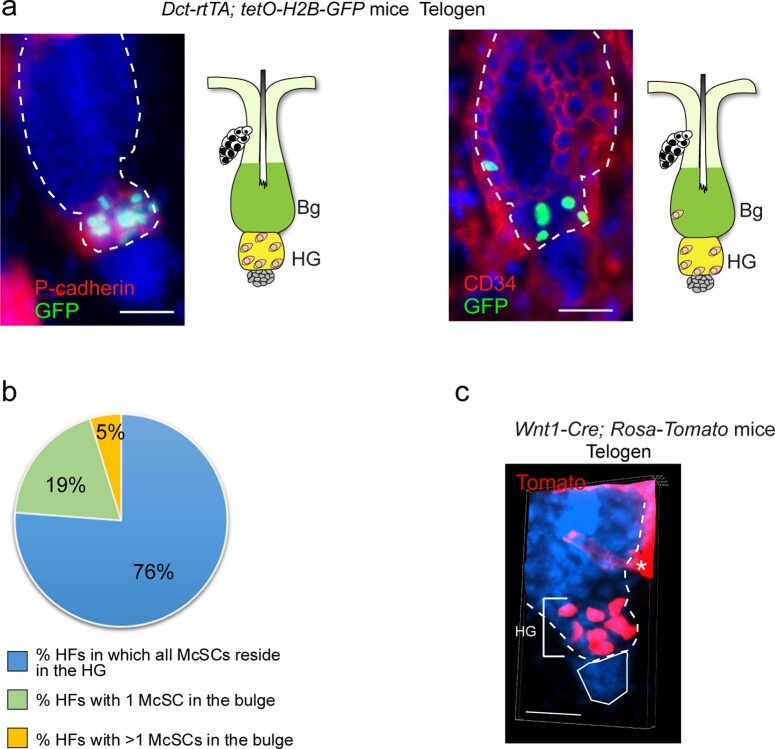
McSCs primarily reside in P-cadherin^+^ HG and occasionally in CD34^+^ bulge at telogen. (**a**) *Z*-stack image of GFP^+^ cells showing P-cadherin or CD34 expression on whole telogen HFs of *Dct-rtTA; tetO-H2B-GFP* mice. Schematics depict observed distribution of GFP^+^ cells. All hair types were included by examining hair follicles without bias per given areas. (**b**) Quantification of the locations of McSCs in the telogen HFs from *Dct-rtTA; tetO-H2B-GFP* mice. HG is considered P-cadherin^+^ and bulge is considered P-cadherin^−^. N = 42 whole HFs from 3 mice. (**c**) Focused image of Tomato^+^ cells in telogen HF of 8 weeks old *Wnt1-Cre; Rosa-LSL-Tomato* mouse (2^nd^ telogen). HG: hair germ; Bg: bulge. Scale bar: 20 μm. Dashed white lines outline epithelial-dermal boundary. Solid circle suggests location of dermal papilla. White asterisk marks Tomato signal outside of the hair follicle. Source data

To better understand the hierarchical structure of the McSC system, we quantified the distribution of McSCs before HF regeneration (that is, in the telogen phase). McSCs are defined as DCT^+^ melanocytes located at the level of the bulge and slightly below the bulge area^4^. DCT is a melanocyte lineage marker expressed by all melanocytes during the hair cycle^4,8,17^. Consistent with previous microscopy analyses^4,5^, DCT^+^ McSCs were found in the bulge and the HG. However, a new volumetric analysis with 3D imaging of whole telogen HFs from *Dct*^*LacZ*^ and *Dct*^*rtTA*^*;tetO*^*H2B-GFP*^ reporter mice^17,18^ revealed that most HFs lacked any McSCs within the CD34^+^ bulge. Rather, most McSCs were concentrated in the P-cadherin^+^ HG (Fig. 1a and Extended Data Fig. 1). Even in the HFs that contained rare bulge McSCs, the majority were located in the HG (Extended Data Fig. 1a). In agreement, labelling of *Wnt1*^*cre*^*;Rosa*^*LSL-tdTomato*^ mice, in which neural-crest-derived cells, including the melanocyte lineage, are labelled by the fluorescent protein tdTomato showed that most tdTomato^+^ McSCs were concentrated in the HG^7^ (Extended Data Fig. 1c). These results suggest that hair pigmentation and McSC maintenance may solely rely on McSCs in the HG.

**Extended Data Fig. 2:**
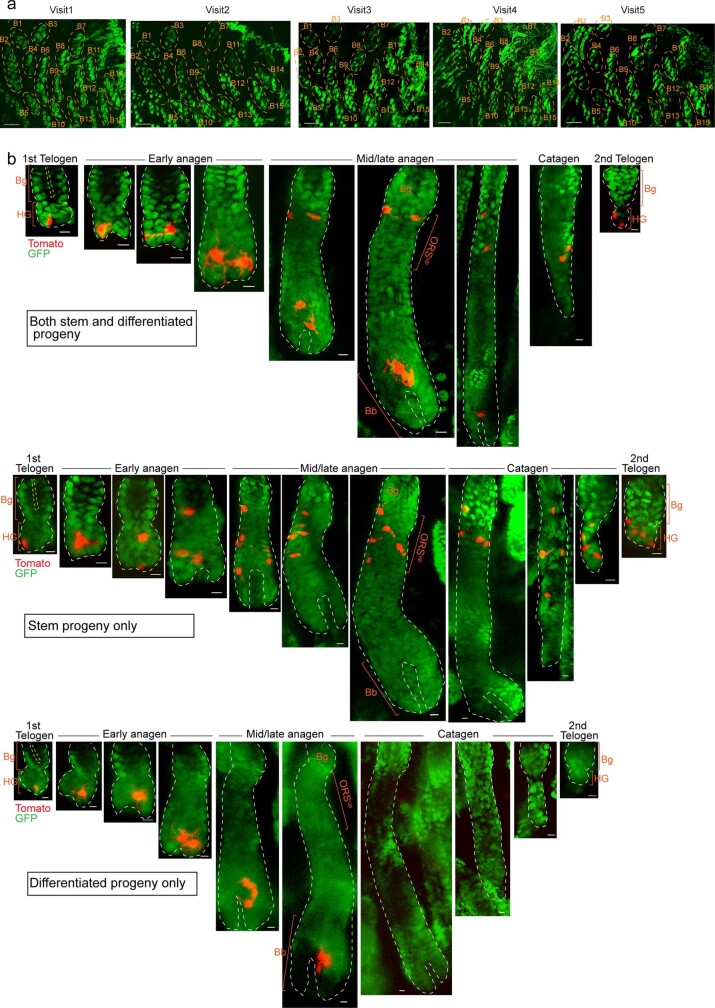
Additional live images of McSC lineage tracing. (**a**) Representative example of large tile images showing the pattern of HF distribution at each visit of the same area to guide repeated accurate tracing of the same HF. Z-stack images show GFP signal (K14^+^ epithelium) in *Tyr-CreER;Rosa-LSL-tdTomato;K14-rtTA;tetO-H2B-GFP* mice. Orange dashed lines mark hair follicle bundles (labeled, B1, B2, B3…). Overlapping patterns were used to identify specific HFs. Shown are representative images, N = 7 mice. (**b**) Additional examples of Fig. 1c. Z-stack images of three independent examples of HFs from *in vivo* imaging of *Tyr-CreER;Rosa-LSL-tdTomato;K14-rtTA;tetO-H2B-GFP* mice imaged as illustrated in Fig. 1b. A single tdTomato-labeled cell within each HF was initially visited at the first telogen stage; then revisited at early anagen, mid/late anagen, catagen and the next telogen (2^nd^ telogen). Shown are representative examples of Tom^+^ HG McSCs that undergo self-renewal and differentiation (top panels), self-renewal (middle panels) and differentiation (bottom panels). In the middle panels, note that one Tom^+^ cell died at the beginning of catagen, so only 4 Tom^+^ cells are present during catagen stages. Yellow dashed line outlines club hair. Dashed white lines outline epithelial-dermal boundary. HG: hair germ; Bb: bulb; Bg: bulge; ORS^up^: upper ORS.

**Extended Data Fig. 3:**
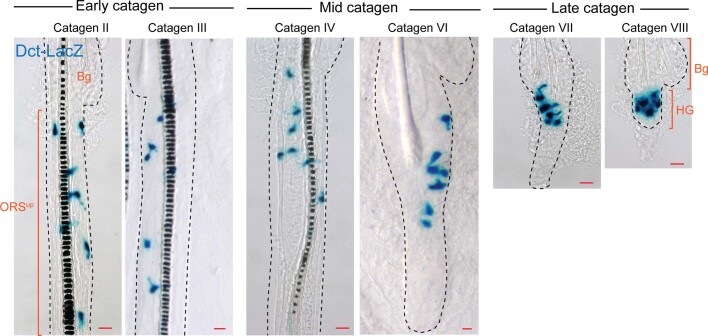
Distribution of McSCs in *Dct-LacZ* mice during catagen stages. Images of hair follicles from *Dct-LacZ* mice at indicated catagen stages. Note that LacZ+ McSCs come together at late catagen to eventually aggregate in the HG. Shown are representative images, N = 5 mice. Dashed black lines outline epithelial-dermal boundary. HG: hair germ; Bg: bulge; ORS^up^: upper ORS. Scale bar: 20 µm.

To verify that HG McSCs have self-renewal ability, we performed fate mapping of telogen HG McSCs using in vivo imaging. We used a mouse model in which McSCs are genetically labelled with tdTomato, and K14^+^ epithelial cells express GFP, which outlines the HF structure (*Tyr*^*creER*^*;Rosa*^*LSL-tdTomato*^*;K14*^*rtTA*^*;tetO*^*H2B-GFP*^ mice^19–22^) (Fig. 1b). *Tyr*^*creER*^ mice are broadly utilized to target melanocytes, including DCT^+^ McSCs^7,8,19,23,24^. For clonal analysis, we treated the mice with a low dose of tamoxifen during telogen and identified HFs with only one labelled McSC in the HG compartment. Revisitation of the same HF during anagen (Methods and Extended Data Fig. 2a) showed that HG McSCs labelled during telogen gave rise to mature melanocytes in the hair bulb that die at the end of anagen^25^ (Fig. 1c–e and Extended Data Fig. 2b). Notably, we also discovered that labelled McSCs migrated to the bulge and upper outer root sheath (bulge/ORS^up^) stem cell niche during anagen and exhibited self-renewal capacity that enabled their persistence (Fig. 1c,d and Extended Data Fig. 2b). During late catagen, most McSCs in the bulge/ORS^up^ were aggregated in the lower follicle (Fig. 1f, Extended Data Fig. 3). By the next telogen phase, they had primarily homed back to the HG compartment (Fig. 1c,g and Extended Data Fig. 2b ). These results demonstrate that unlike the HFSC system, in which HG epithelial cells disappear after proliferation and differentiation^15,26^, melanocytes residing within the HG possess self-renewal ability.

## Trajectory of McSC differentiation at anagen onset

Previous studies have shown that WNT proteins in the HG promote differentiation of epithelial cells and melanocytes following induction of HF regeneration^7^. Epithelial HG cells then function as TA cells that proliferate and differentiate without self-renewal capacity^15,26^. To investigate how McSCs can retain their stemness while being aggregated in such a pro-differentiation environment, we examined the differentiation status of HG McSCs.

**Fig. 2:**
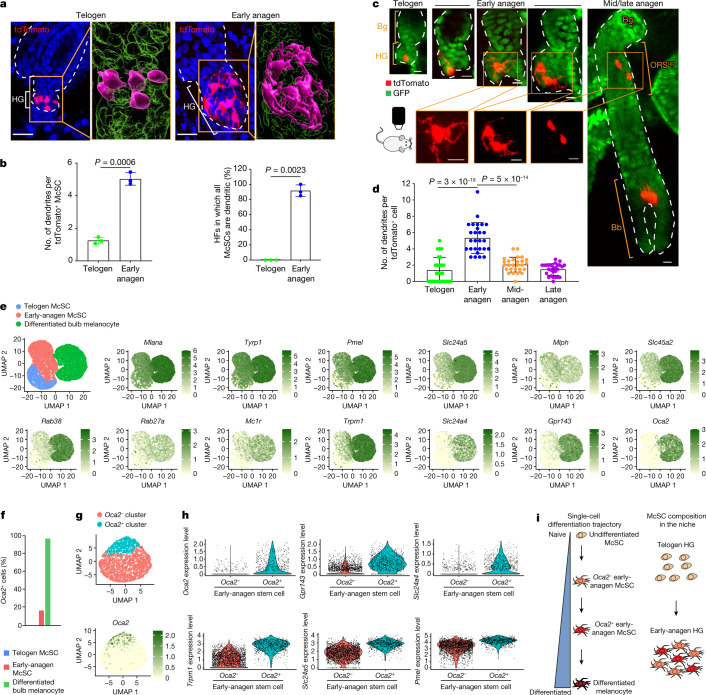
McSCs in the HG initiate a differentiation programme during early anagen. **a**, *z*-stack images of tdTomato^+^ McSCs from *Tyr*^*creER*^*;Rosa*^*LSL-tdTomato*^ mice at telogen and early anagen. Denoted areas are magnified and reconstructed using Imaris (magenta, tdTomato^+^; green, DAPI). **b**, Left, number of dendrites per tdTomato^+^ McSCs (*N* = 3 mice, ≥30 tdTomato^+^ McSCs analysed per mouse). Right, percentages of HFs containing only dendritic McSCs (*N* = 3 mice, 20 HFs analysed per mouse). Melanocytes with ≥3 dendrites were considered dendritic. Data are presented as the mean ± s.d. *P* values derived by two-tailed unpaired *t*-test. **c**, Live revisits of a representative first telogen HF containing a single HG tdTomato-labelled cell in a *Tyr*^*CreER*^*;Rosa*^*LSL-tdTomato*^*;K14*^*rtTA*^*;tetO*^*H2B-GFP*^ mouse. K14^+^ epithelial cells (GFP^+^) are in green. Bottom panels show magnified views. **d**, Dendrite number per tdTomato^+^ McSCs in HG (telogen and early anagen) and bulge/ORS^up^ (mid-anagen and late anagen). *N* = 27 HFs from 2 mice. Data are presented as the mean ± s.d. *P* values derived using one-way ANOVA with Bonferroni multiple comparison test, with the following 95% confidence intervals: −4.804 to −3.122 (telogen versus early anagen); 2.430 to 4.112 (early anagen versus mid-anagen). **e**, Uniform manifold approximation and projection (UMAP) plot of merged FACS-isolated telogen McSCs, early-anagen McSCs and differentiated anagen VI bulb melanocytes. FeaturePlots show expression of pigmentation genes. **f**, Percentages of *Oca2*^+^ cells within each population. **g**, UMAP plot of early-anagen McSCs after regression of cell cycle genes. The FeaturePlot shows *Oca2* expression. **h**, Violin plots showing differential expression of pigmentation genes in early-anagen *Oca2*^+^ and *Oca2*^−^ clusters. **i**, Schematic of melanocyte differentiation trajectory and McSC composition in the niche. Dashed white lines outline the epithelial–dermal boundary (**a**,**c**). Scale bars, 20 μm (**a**) or 10 μm (**c**). Source data

**Extended Data Fig. 4:**
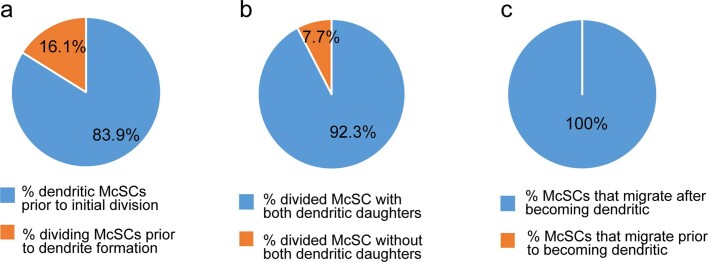
Quantification of dendrite state of McSCs by live imaging. Quantification of live imaging experiment in Fig. 2c. (**a**) Percentages of dendritic McSCs prior to or after initial division. N = 31 HFs from 2 mice. (**b**) Percentages of post-mitotic McSCs with or without both dendritic daughters. N = 26 HFs from 2 mice. (**c**) Percentages of McSCs that migrate before or after dendrite formation. N = 35 HFs from 2 mice. Source data

In the quiescent telogen stage, McSCs displayed the compact oval or bipolar shape of undifferentiated melanocytes. However, they showed marked changes during early anagen (anagen II), when TA cells emerge in this HG compartment^4–6^, with all melanocytes in the HG developing a dendritic appearance reminiscent of differentiated melanocytes^27^ (Fig. 2a,b). In more than 90% of the HFs examined, all melanocytes had dendritic morphology during this phase (Fig. 2b). Live imaging showed that the majority of HG McSCs had transformed into a dendritic morphology within the niche before their initial division (Fig. 2c and Extended Data Fig. 4). Daughter cells were also dendritic, which suggested that morphological changes represent an early feature of McSC activation (Fig. 2c and Extended Data Fig. 4). This morphological change was transient, and McSCs retracted dendrites after they migrated from the HG to the bulge/ORS^up^ (Fig. 2c,d).

**Extended Data Fig. 5:**
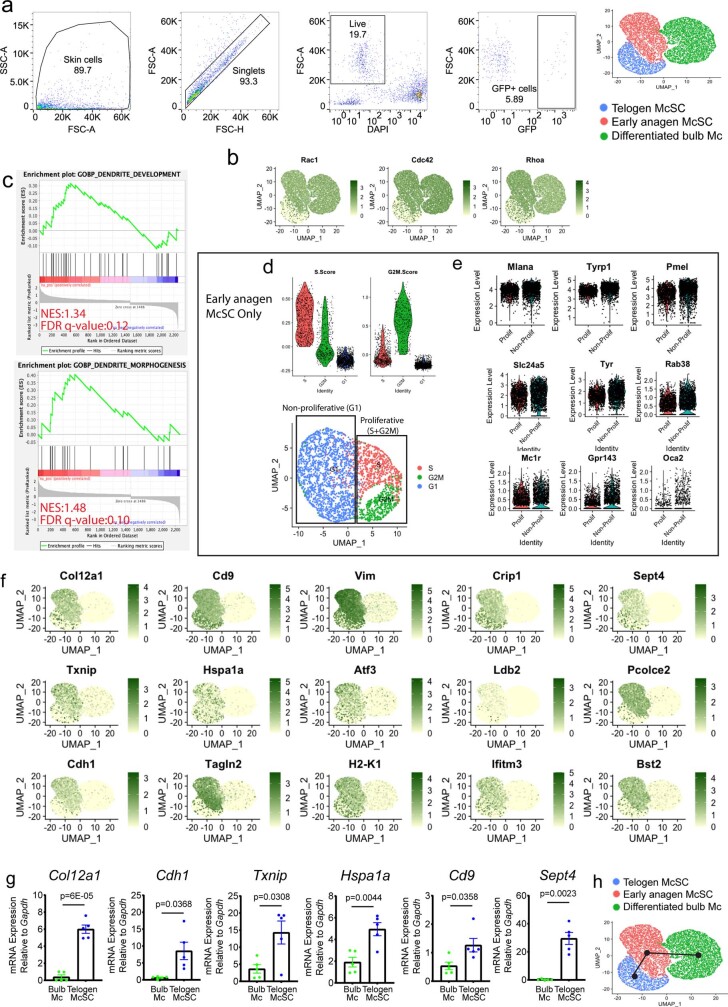
Early anagen McSCs activate dendrite and pigmentation related genes while retaining the expression of SC-enriched genes. (**a**) FACS plots illustrating the gating strategy to isolate GFP^+^ melanocytes from *Dct-rtTA; tetO-H2B-GFP* mice. UMAP plot of merged scRNA-seq results from sorted telogen McSCs (blue), anagen II McSCs (Early anagen McSC, red) and anagen VI bulb Mcs (Differentiated bulb Mc, green). (**b**) Featureplots showing regions and levels of expression of indicated dendrite-related genes. (**c**) GSEA of Gene Ontology Biological Process (GOBP) dendrite development and dendrite morphogenesis in differentially expressed genes (DEGs) from early anagen McSCs compared to telogen McSCs. (**d**) Top, violin plots of the cell cycle scores of early anagen McSCs. Bottom, UMAP plot of early anagen McSCs clustered by their cell-cycle score. The S and G2M phase cells are grouped together into proliferative early anagen McSCs and the G1 phase cells constitute the non-proliferative early anagen McSCs. (**e**) Violin plots showing expression of pigmentation genes in the proliferative (Prolif) and non-proliferative (Non-Prolif) early anagen McSCs. (**f**) Featureplots showing expression of indicated SC-enriched genes in telogen McSCs, early anagen McSCs and late anagen bulb Mcs. (**g**) Quantitative RT-PCR analysis of indicated SC-enriched genes in bulb Mcs and telogen McSCs. N = 5 mice. Data are represented as mean ± SEM. P-values by two-tailed unpaired t test. (**h**) UMAP plot with dots and lines showing pseudo-time trajectory predicted using slingshot package. Source data

Single-cell RNA sequencing (scRNA-seq) comparisons of telogen and early-anagen McSCs and mature bulb melanocytes purified by fluorescence-activated cell sorting (FACS) verified that genes involved in melanocyte dendrite formation^28,29^, such as *Rac1*, were upregulated in early anagen, similar to that observed in mature bulb melanocytes of anagen HFs (Extended Data Fig. 5a–c). Given that the definition of differentiation in biology is “a process by which a less specialized cell matures to fulfil the function of the lineage”^30^, we examined pigmentation genes as reliable differentiation markers. FeaturePlot analysis showed that numerous pigmentation genes, absent from telogen McSCs, were detected in most early-anagen McSCs (Fig. 2e). Furthermore, a comparison of proliferative and nonproliferative early-anagen McSCs revealed similar signatures, which suggested that differentiation may occur independent of the proliferative state (Extended Data Fig. 5d,e). Nonetheless, a distinct set of genes was expressed by both telogen and early-anagen HG McSCs, but downregulated in bulb melanocytes (Extended Data Fig. 5f,g). Some of these genes have potential stem cell relevance, with functions related to renewal and survival^31,32^. Pseudotime analysis confirmed that early-anagen McSCs reside between telogen McSCs and bulb melanocytes (Extended Data Fig. 5h).

## The most differentiated McSCs can dedifferentiate 

On the basis of these analyses, we formulated the following hypothesis. McSCs may differentiate into an intermediate differentiation (TA-like) state at the whole population level in the growing HG following cues of regenerative stimuli, and McSC maintenance may rely on their dedifferentiation.

**Extended Data Fig. 6:**
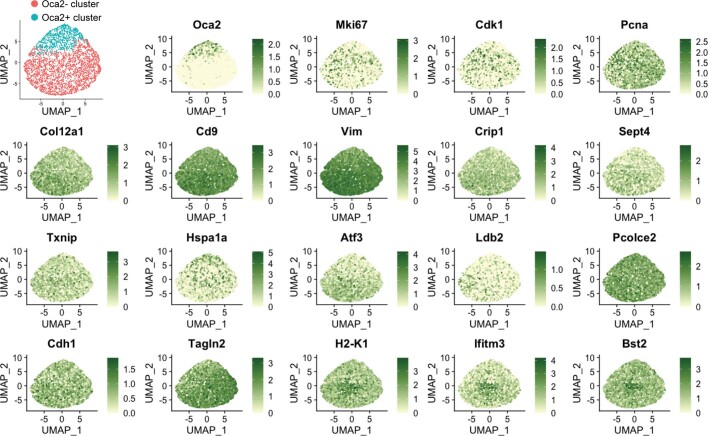
Examination of the early anagen HG scRNAseq dataset for proliferation and expression of SC-enriched genes. UMAP of early anagen McSCs after cell cycle genes regression. Featureplots of proliferation genes and SC-enriched genes in Oca2^−^ and Oca2^+^ early anagen McSCs.

To test this hypothesis, we sought to trace the fate of the most differentiated subset of HG McSCs. We focused on a late-pigmentation gene, *Oca2* (refs. ^33–35^), that is not expressed by quiescent telogen McSCs but is upregulated in all mature bulb melanocytes based on our scRNA-seq data (Fig. 2e,f). Notably, a small subset of early-anagen McSCs also expressed *Oca2* (Fig. 2e,f). To confirm that *Oca2*^+^ McSCs are more differentiated than *Oca2*^–^ McSCs in early-anagen HFs, we reanalysed scRNA-seq data in which *Oca2*^+^ cells had diverged from other early-anagen McSCs by regressing out cell cycle genes (Fig. 2g). Analyses revealed that differentiation markers (that is, pigmentation genes) were expressed at higher levels in *Oca2*^+^ than in *Oca2*^–^ McSCs (Fig. 2h). Unlike *Oca2*^+^ bulb melanocytes, the *Oca2*^+^ early-anagen McSCs retained expression of genes shared by telogen and other early-anagen McSCs (Extended Data Fig. 6). These results suggest that whereas most HG melanocytes at the early-anagen phase initiate the upregulation of differentiation and pigmentation genes, the most differentiated ones also express *Oca2* (Fig. 2i).

**Fig. 3:**
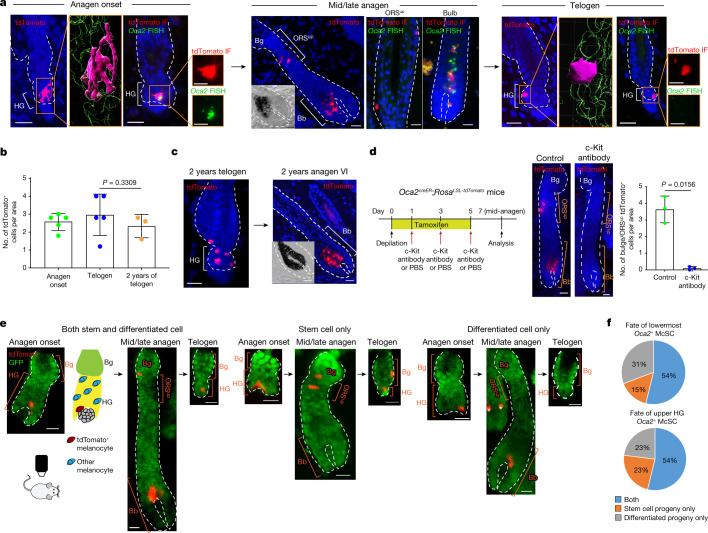
*Oca2*^+^ cells located in the TA compartment can undergo dedifferentiation during anagen. **a**–**c**, *Oca2*^*creER*^*;Rosa*^*LSL-tdTomato*^ mice were injected with tamoxifen three times during depilation-induced anagen onset for *Oca2*^+^ cell lineage tracing. **a**, Detection of tdTomato and *Oca2* (fluorescence in situ hybridization (FISH)) as indicated. Denoted areas of tdTomato-only detection are reconstructed using Imaris (magenta, tdTomato^+^; green, DAPI). Magnified views of tdTomato immunofluorescence (IF) and OCA2 FISH are represented in single colour. **b**, Number of tdTomato^+^ cells over time. *N* = 5 mice (anagen onset and telogen) and *N* = 3 mice (2-year telogen). Five areas were analysed per mouse. *P* values were derived using one-way ANOVA with Bonferroni multiple comparison test, with 95% confidence intervals of −0.7397 to 1.993. **c**, Detection of tdTomato in telogen and induced anagen at 2 years following tamoxifen treatment. **d**, Left, timeline of injections and analysis 7 days after depilation of tdTomato expression in *Oca2*^*creER*^*;Rosa*^*LSL-tdTomato*^ mice treated with PBS (control) or a c-Kit-neutralizing antibody. Right, images and quantification of tdTomato^+^ McSCs. *N* = 3 mice. Ten areas were analysed per mouse. *P* value derived by two-tailed unpaired *t*-test. **e**, Live lineage tracing of a single tdTomato^+^ cell of *Oca2*^*creER*^*:Rosa*^*LSL-tdTomato*^*;K14*^*rtTA*^*:tetO*^*H2B-GFP*^ mice reveals three fates. GFP marks K14^+^ epithelial cells. Cartoon illustrates the relative locations of tdTomato^+^ melanocytes and other melanocytes in the HF. White asterisks indicate tdTomato^+^ cells in an unrelated HF. **f**, Percentages of single *Oca2*^+^ cells in the lowest HG region (*N* = 13 HFs from 1 mouse) or the upper HG (*N* = 13 HFs from 1 mouse) that give rise to specific progeny as defined. Insets in mid/late anagen images in **a** and **c** show bright-field images of the bulb. For **b** and **d**, data are presented as the mean ± s.d. Scale bars, 20 μm or 10 μm (single colour images of **a**). Dashed white lines outline the epithelial–dermal boundary (**a**,**c**,**d**,**e**). Source data

**Extended Data Fig. 7:**
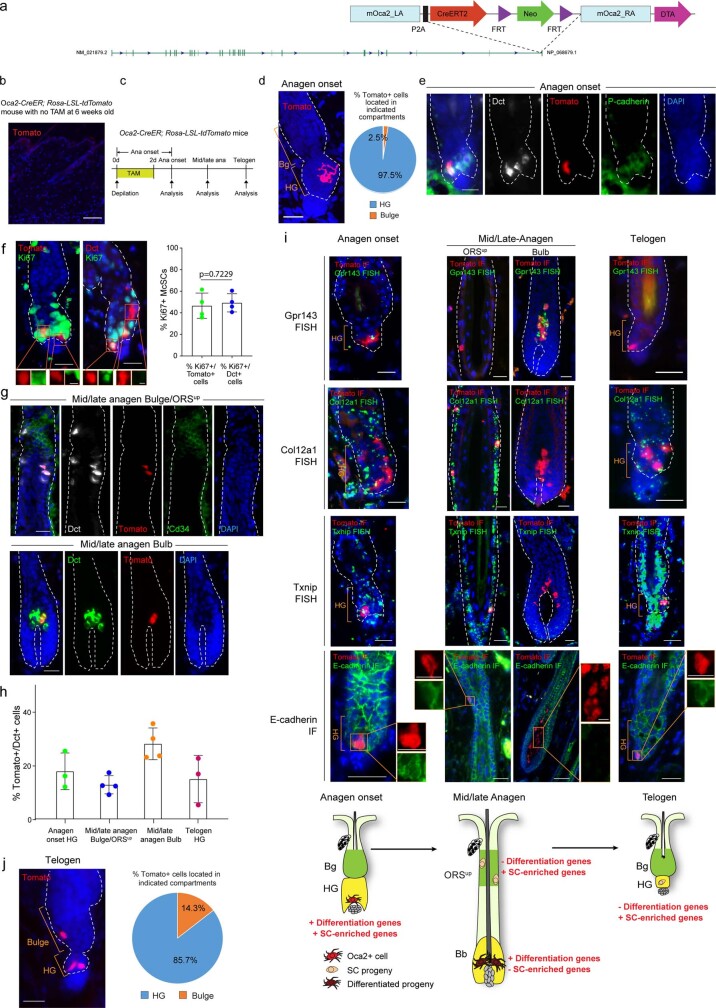
Characterization of Tomato^+^ cells in *Oca2-CreER;Rosa-LSL-tdTomato* mice during lineage tracing. (**a**) Targeting vector design of *Oca2-CreERT2* knock-in mouse. (**b**) Cryo section image of *Oca2-CreER;Rosa-LSL-tdTomato* mouse back skin without TAM treatment at 6–13 weeks old. Shown is representative image of 4 mice. (**c**) Experimental design for panels (**d**)-(**j**). Lineage tracing of Oca2^+^ HG McSCs in *Oca2-CreER; Rosa-LSL-tdTomato* HFs. (**d**) Z-stack image of Oca2^+^ (Tom^+^) cell in whole hair follicle at anagen (Ana) onset. Analysis of the location of Oca2^+^ (Tom^+^) cells in indicated niche compartments at anagen onset. N = 4 mice with at least 10 HFs analyzed per mice. (**e**) Merged or separate immunofluorescence images of Dct (white), Tomato (red), P-cadherin (green) and DAPI (blue) at early anagen. (**f**) Immunofluorescence of Tomato and Ki67 and Dct and Ki67 in early anagen. Lower panels depict magnified single color images in specified regions. Quantification of Ki67^+^/Tomato^+^ cells or Ki67^+^/Dct^+^ cells. N = 4 mice in each group with at least 10 HFs analyzed per mouse. Data are represented as mean ± SD. P-values by two-tailed unpaired t test. (**g**) Top: Merged or separate immunofluorescence images of Dct (white), Tomato (red), Cd34 (green) and DAPI (blue) in mid/late anagen bulge/ORS^up^. Bottom: Merged or separate immunofluorescence images of Dct (green), Tomato (red) and DAPI (blue) in mid/late anagen bulb. (**h**) Quantification of Tomato^+^/Dct^+^ cells. N = 3 mice in anagen onset HG and telogen HG groups and N = 4 mice in mid/late anagen bulge/ORS^up^ and bulb groups with at least 10 HFs analyzed per mouse per compartment. Data are represented as mean ± SD. (**i**) Immunofluorescence of tdTomato and *in situ* hybridized Gpr143 probe, Col12a1 probe and Txnip probe. Col12a1 and Txnip were also observed in unrelated epithelial cells. IF shows tdTomato and e-cadherin staining with boxed single color enlargements. Schematic illustration of differentiation genes (Oca2, Gpr143) and SC-enriched genes (Col12a, Txnip, E-Cadherin) expression in an early anagen Oca2+ cell and its progeny at subsequent hair cycle stages. Shown are representative images, N = 3 mice. (**j**) Z-stack image of trace labeled Tom^+^ cells in subsequent telogen. Quantification of the location of Tom^+^ cells in indicated niche compartments in subsequent telogen. N = 3 mice with at least 5 HFs analyzed per mice. Dashed white lines outline epithelial-dermal boundary. Scale bar: 20 μm, 10 µm in separate channel images. IF: immunofluorescence; FISH: fluorescence *in situ* hybridization; HG: hair germ; Bb: bulb; Bg: bulge; ORS^up^: upper ORS; TAM: tamoxifen; d: day. Source data

We then generated *Oca2*^*creER*^*;Rosa*^*LSL-tdTomato*^ reporter mice as a means to genetically trace the fate of *Oca2*^+^ HG McSCs (Extended Data Fig. 7a). No tdTomato^+^ cells were observed in the HFs of mice that did not receive a tamoxifen injection (Extended Data Fig. 7b). Using this new tool, we traced the fate of McSCs from anagen onset onwards. We treated *Oca2*^*creER*^*;Rosa*^*LSL-tdTomato*^ mice with tamoxifen once daily for 3 days during early anagen (Extended Data Fig. 7c). This treatment successfully labelled *Oca2*^+^ (tdTomato-labelled) McSCs, which displayed dendritic morphology, expressed *Oca2* mRNA and were found in the HG (Fig. 3a and Extended Data Fig. 7d,e). *Oca2*^+^ cells included both proliferative and nonproliferative McSCs (Extended Data Fig. 7f), results that are consistent with our scRNA-seq data (Extended Data Fig. 5d,e).

Once the HF fully formed the anagen bulb, cells initially labelled for *Oca2* (tdTomato^+^) in the HG had contributed to both differentiated melanocytes in the hair bulb and to McSCs in the anagen bulge/ORS^up^ (Fig. 3a and Extended Data Fig. 7g,h). By mid-to-late (mid/late) anagen, tdTomato^+^ cells within the bulge/ORS^up^ niche reverted to an *Oca2*^–^ state (Fig. 3a), and other differentiation markers (that is, pigmentation genes) such as *Gpr143* were downregulated (Extended Data Fig. 7i).

By the subsequent telogen phase, most tdTomato^+^ cells had relocated to the HG (Extended Data Fig. 7j) and retained their undifferentiated state (Fig. 3a). These tdTomato^+^ McSCs retained the ability to regenerate bulb melanocytes for at least 2 years (the final time point examined; Fig. 3b,c). In agreement with our scRNA-seq data (Extended Data Figs. 5f and 6), those HG *Oca2*^+^ McSCs destined for dedifferentiation retained their expression of stem-cell-enriched genes (for example, *Col12a1*, *Txnip* and *Cdh1* (which encodes E-cadherin)), whereas those destined for final maturation in the bulb lost expression of these genes (Extended Data Fig. 7i).

**Extended Data Fig. 8:**
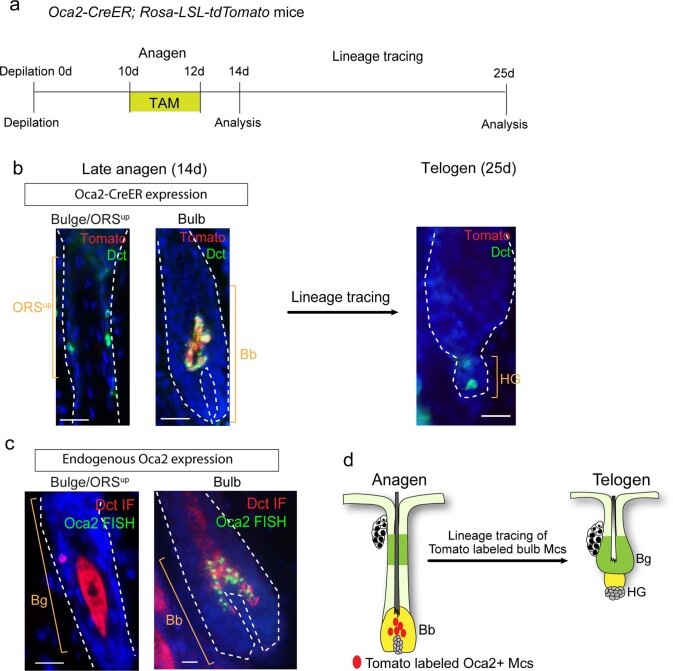
Genetic lineage tracing shows that Oca2^+^ bulb Mcs do not home back to the HG. (**a**) Experimental design for lineage tracing of Oca2^+^ late anagen bulb Mcs in *Oca2-CreER;Rosa-LSL-tdTomato* HFs. (**b**) Left, immunofluorescence images showing Dct and Tomato (Oca2) expression in McSCs and Mcs in late anagen (depilation 14d) bulge/ORS^up^ and bulb. Right (after arrow), immunofluorescence image showing lack of Tomato labeling in Dct^+^ McSCs at subsequent telogen (depilation 25d). Shown are representative images, N = 3 mice. (**c**) Immunofluorescence images of Dct and *in situ* transcriptional localization of endogenous Oca2 mRNA in late anagen bulge/ORS^up^ and bulb from TAM-untreated mice. Shown are representative images of 3 mice. Note that the pattern of endogenous Oca2 mRNA is consistent with *Oca2-CreER* activity (Tomato signals) in (**b**) left panels. (**d**) Schematic illustration of the fate of Tomato labeled bulb Mcs. Dashed white lines outline epithelial-dermal boundary. IF: immunofluorescence; FISH: fluorescence *in situ* hybridization. HG: hair germ; Bg: bulge; Bb: bulb; ORS^up^: upper ORS; TAM: tamoxifen; d: day. Scale bar: 20 μm.

Lineage tracing of late-anagen *Oca2*^+^ differentiated bulb melanocytes confirmed the live-imaging results that bulb melanocytes do not persist (Extended Data Fig. 8). These results demonstrate that highly differentiated *Oca2*^+^ McSCs in the HG but not mature bulb melanocytes can dedifferentiate to give rise to undifferentiated McSCs in the niche.

## McSCs in the TA compartment can dedifferentiate

Previous studies have shown that TA cells in the McSC system depend on c-Kit and can be eliminated after injection of a c-Kit-neutralizing antibody^4,6,36^. To determine whether *Oca2*^+^ cells depend on c-Kit, we injected a c-Kit-neutralizing antibody into mice. This resulted in almost complete elimination of tdTomato^+^ cells (Fig. 3d), which suggested that TA cells that emerge in early anagen include *Oca2*^+^ McSCs. Current theories indicate that such TA cells are spatially segregated in the lower part of the HG during early anagen^5–8^. We used in vivo live imaging to verify whether *Oca2*^+^ McSCs in this area could still revert to a stem cell state. We identified HFs in which a single *Oca2*^+^ McSC was genetically labelled in the lowermost portion of the growing HG and bulb (in anagen II/IIIa HFs of *Oca2*^*creER*^*;Rosa*^*LSL-tdTomato*^*;K14*^*rtTA*^*;tetO*^*H2B-GFP*^ mice). Revisiting these HFs at mid/late anagen revealed that *Oca2*^+^ McSCs in the lower HG were able to translocate to the bulge/ORS^up^, the stem cell compartment of the HF and relocated to the HG by the next telogen, similar to upper HG *Oca2*^+^ McSCs (Fig. 3e,f). Alternatively, they could also descend with HF downgrowth and mature into fully differentiated melanocytes in the bulb (Fig. 3e,f). These results demonstrate that late-stage *Oca2*^+^ McSCs positioned in the lowermost part of the growing HG, known as the TA compartment, can still dedifferentiate and persist long term.

## No McSC is inherently exempted from differentiation

**Extended Data Fig. 9:**
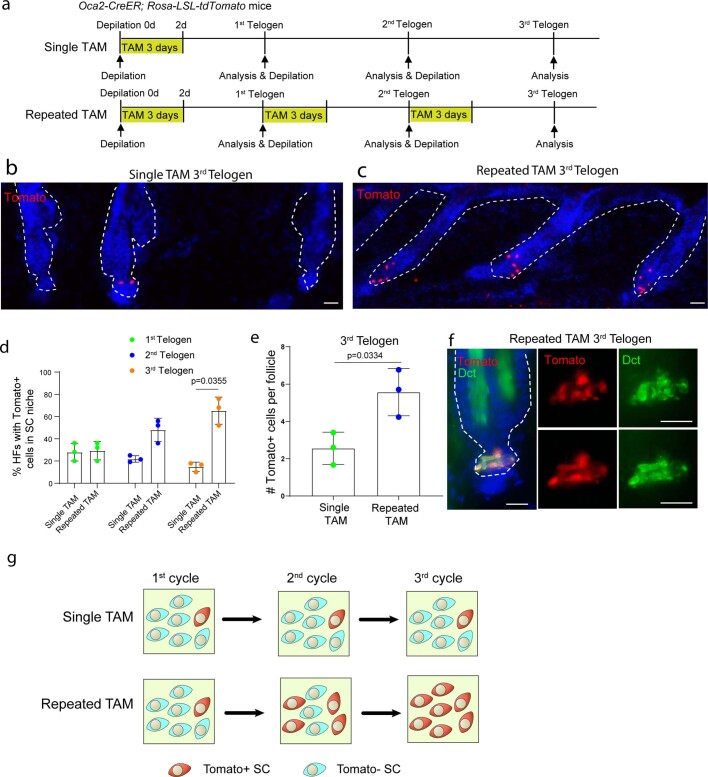
New McSCs become Oca2^+^ at each hair cycle. (**a**) Experimental scheme of TAM injection during one hair cycle (single TAM) or several continuous hair cycles (repeated TAM) in *Oca2-CreER;Rosa-LSL-tdTomato* mice. (**b**-**c**) Z-stack images of trace-labeled Tom^+^ cells in 3^rd^ telogen HFs after single (**b**) or repeated (**c**) TAM injections. (**d**) Number of HFs containing Tom^+^ cells in HG after single or multiple TAM injections. HFs were counted at 1^st^ telogen (green), 2^nd^ telogen (blue) and 3^rd^ telogen (orange). N = 3 mice, with at least 25 HFs analyzed per mice per time point. Data are represented as mean ± SD. P-value by two-way ANOVA with Bonferroni multiple comparison test. 95% confidence interval: −94.18 to −6.799. (**e**) Number of Tomato^+^ McSC per hair follicle from single and multiple TAM-injected mice at 3^rd^ telogen. N = 3 mice with at least 3 HFs containing Tom+ cells analyzed per mice. Data are represented as mean ± SD. P-value by two-tailed unpaired t test. (**f**) Z-stack image of Tomato and Dct co-immunofluorescence at 3^rd^ telogen following repeated TAM injections. To show the co-localization of green and red signals in all McSCs throughout the z-stack, the right top panels show the z-stack of first 15 stacks and the right bottom panels show the z-stack of last 15 stacks. Shown are representative images of 3 mice. (**g**) Schematic summary. Dashed white lines outline epithelial-dermal boundary. Scale bar: 20 μm. TAM: tamoxifen; d: day. Source data

As not all McSCs express *Oca2* during the early-anagen phase, we asked whether the *Oca2*^+^ subset might constitute a fixed subpopulation in the HF or whether other McSCs can also express *Oca2* during a subsequent anagen phase. To test this theory, we repeatedly induced HF regeneration and labelled *Oca2*^+^ McSCs at each anagen onset through tamoxifen treatment (Extended Data Fig. 9a). We observed gradual increases in the percentage of HFs containing tdTomato^+^ cells and in the number of tdTomato^+^ McSCs per HF (Extended Data Fig. 9b–e). This result contrasted with control experiments, in which *Oca2*^+^ McSCs were labelled only once. These results show that new (unlabelled) McSCs can express *Oca2* at different anagen onsets (approximately 20% at one hair cycle based on scRNA-seq analysis) and contribute to both fates. After three cycles of repeated tamoxifen injection, HF niches in which residents were exclusively tdTomato^+^ McSCs (>20% HFs analysed) were observed (Extended Data Fig. 9f), which demonstrated that different McSCs express *Oca2* in different hair cycles. These results suggest that there is no reserved population of stem cells that are inherently exempt from experiencing *Oca2*^+^ TA-like status (Extended Data Fig. 9g).

## Pigmented McSCs can dedifferentiate

**Extended Data Fig. 10:**
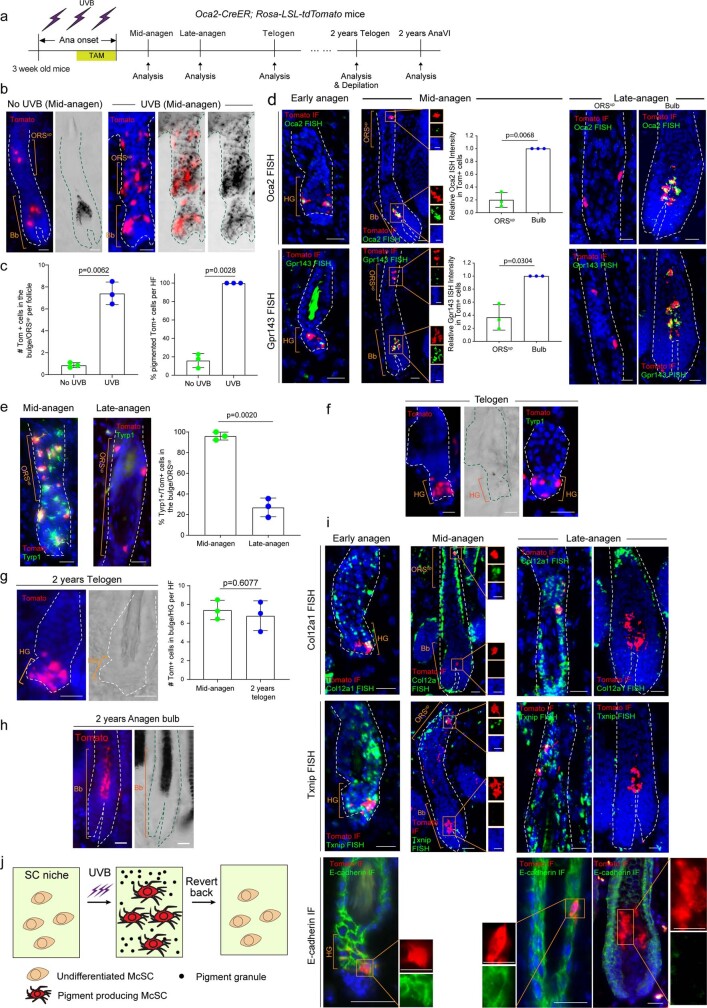
Reversible differentiation of pigmented McSCs following UVB irradiation. (**a**) Experimental scheme: *Oca2-CreER;Rosa-LSL-tdTomato* mice were irradiated with UVB for 3 times and injected with TAM for 3 times during anagen onset and analyzed at the following time points. (**b**) *Z*-stack and corresponding brightfield images (separate and merged) of Tom^+^ cells in mid-anagen control and UVB-treated HFs. (**c**) Number of Tom^+^ cells in bulge/ORS^up^ per HF; percentages of pigmented Tom^+^ cells in bulge/ORS^up^ per HF at mid-anagen. N = 3 mice. 20 HFs analyzed per mice. (**d**) Immunofluorescence of tdTomato and *in situ* hybridized Oca2 probe and Gpr143 probe. Quantification of the FISH intensity of Oca2 or Gpr143 probe expression in Tomato^+^ cells in ORS^up^ relative to the FISH intensity of Tomato^+^ cells in the bulb analyzed by HALO FISH module. N = 3 mice with at least 20 Tom^+^ cells analyzed per compartment per mouse. (**e**) Immunofluorescence images show localization of tdTomato and differentiation marker Tyrp1 at indicated time points. Percentages of Tyrp1^+^/Tom^+^ cells in the bulge/ORS^up^. N = 3 mice. 10 HFs analyzed per mouse. (**f**) Z-stack image of Tom^+^ cells with corresponding brightfield and Tyrp1 staining for UVB-treated mice at subsequent telogen. Shown are representative images, N = 3 mice. (**g**) *Z*-stack image of trace-labeled Tom^+^ cells and corresponding brightfield image in telogen HF from UVB-treated mouse at 2 years. Number of Tom^+^ cells per hair follicle after UVB irradiation at mid-anagen and 2 years. N = 3 mice with 20 HFs analyzed per mice. (**h**) *Z*-stack image of trace-labeled Tom^+^ cells and corresponding brightfield image in anagen VI HF bulb region from UVB-treated mouse at 2 years. (**i**) Immunofluorescence of tdTomato and *in situ* hybridized Col12a1 probe and Txnip probe. IF shows tdTomato and e-cadherin staining with boxed single color enlargements. Shown are representative images, N = 3 mice. (**j**) Schematic postulates that all McSCs can undergo differentiation to make pigment after UV treatment, followed by reversion to an undifferentiated state, then self-renewal and regeneration of differentiated Mcs over time. Dashed white lines outline epithelial-dermal boundary. Scale bar: 20 μm, 10 µm in separate channel images. Data are represented as mean ± SD in (**c**), (**d**), (**e**) and (**g**). P-values by two-tailed unpaired t test in (**c**), (**d**), (**e**) and (**g**). IF: immunofluorescence; FISH: fluorescence *in situ* hybridization; HG: hair germ; Bb: bulb; ORS^up^: upper ORS; TAM: tamoxifen. Source data

Given that the skin is constantly exposed to environmental factors that promote melanocyte differentiation^37,38^, we asked whether McSCs can still dedifferentiate in such an environment. To that end, we irradiated *Oca2*^*creER*^*;Rosa*^*LSL-tdTomato*^ mice with UVB light and injected tamoxifen to label *Oca2*^+^ cells with the tdTomato reporter (Extended Data Fig. 10a). Two days after treatment, tdTomato^+^ McSCs produced abundant pigment in the stem cell niche (Extended Data Fig. 10b,c). UVB-treated McSCs expanded (Extended Data Fig. 10b,c) and expressed differentiation markers in the mid-anagen bulge/ORS^up^, albeit to a lesser extent compared with bulb melanocytes (Extended Data Fig. 10d). These results suggest that UVB irradiation induces McSCs to accelerate their differentiation into functional melanocytes. By late anagen, tdTomato^+^ McSCs located in the bulge/ORS^up^ had significantly downregulated expression of the differentiation markers *Tyrp1*, *Oca2* and *Gpr143*, which suggested that they were undergoing dedifferentiation (Extended Data Fig. 10d,e). By the next telogen phase, tdTomato^+^ McSCs still homed back to the HG. They regained an undifferentiated phenotype, as evidenced by the downmodulation of differentiation markers and pigmentation (Extended Data Fig. 10f). Two years after UVB irradiation, comparable numbers of tdTomato^+^ McSCs were observed in the HG and pigment was absent (Extended Data Fig. 10g). tdTomato^+^ McSCs remained competent to generate bulb MCs capable of hair pigmentation following depilation-induced HF regeneration (Extended Data Fig. 10h). As observed for untreated *Oca2*^+^ McSCs (Extended Data Fig. 7i), the UVB-treated *Oca2*^+^ McSCs destined for dedifferentiation retained expression of stem-cell-enriched genes (for example, *Col12a1*, *Txnip* and E-cadherin) (Extended Data Fig. 10i). These results show that McSCs that are driven to fulfil their ultimate differentiated function of producing visible pigment following UVB irradiation can still revert to an undifferentiated state (Extended Data Fig. 10j).

## Dynamic niche regulates McSC reversibility

**Extended Data Fig. 11:**
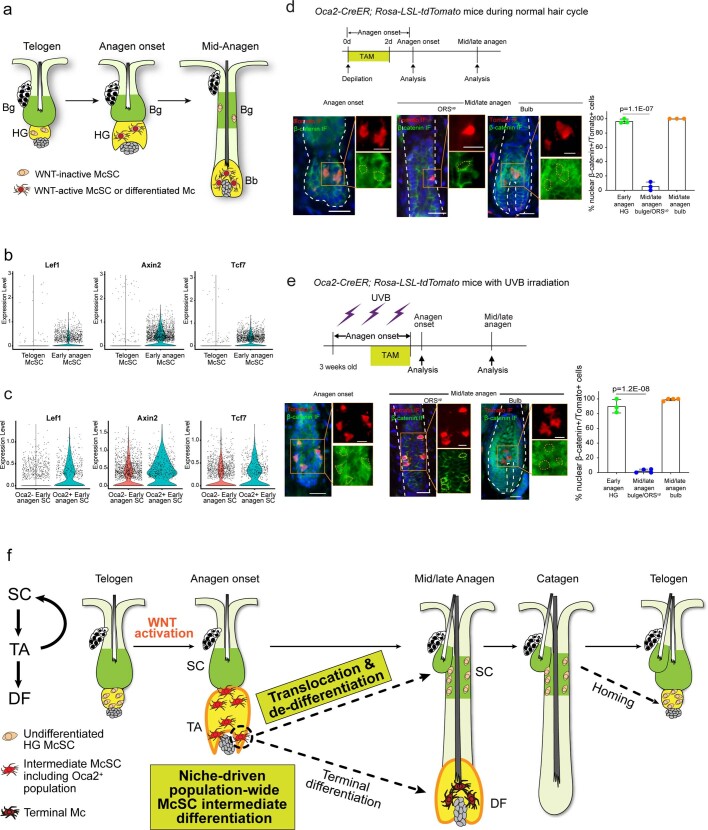
McSCs reversibly enter distinct differentiation states correlating with WNT signaling. (**a**) WNT activation pattern in McSCs as previously reported^7^. (**b**) VlnPlots show expression of WNT genes in telogen McSCs and early anagen McSCs. (**c**) VlnPlots show expression of WNT genes in Oca2^+^ early anagen McSCs and Oca2^–^ early anagen McSCs. For (**b**), see Fig. 2e for related UMAP. For (**c**), see Fig. 2g for related UMAP. (**d**) Experimental scheme of *Oca2-CreER;Rosa-LSL-tdTomato* mice during normal hair cycle. Double-detection of tdTomato and β-catenin in normal hair cycle shown with enlarged single-color images of the denoted areas. Percentages of nuclear β-catenin^+^Tomato^+^ cells. N = 3 mice with at least 10 HFs analyzed per compartment per mouse. Data are represented as mean ± SD. P-values by ordinary one-way ANOVA with Bonferroni multiple comparison test. 95% confidence intervals: 83.22 to 98.56. (**e**) Experimental scheme of *Oca2-CreER;Rosa-LSL-tdTomato* mice with UVB irradiation. Double-detection of tdTomato and β-catenin with UVB treatment shown with enlarged single-color images of the denoted areas. Percentages of nuclear β-catenin^+^Tomato^+^ cells. N = 3 mice in early anagen HG and N = 4 mice in mid/late anagen bulge/ORS^up^ and bulb groups with at least 10 HFs analyzed per compartment per mouse. Data are represented as mean ± SD. P-values by ordinary one-way ANOVA with Bonferroni multiple comparison test. 95% confidence intervals: 79.21 to 96.66. (**f**) Schematic summary of reversible differentiation model. McSCs in the HG during the quiescent telogen phase are in a stem-like state. At anagen onset (regeneration initiation), they undergo activation and differentiation to a TA-like (intermediate) state via WNT activation. These cells then either fully differentiate into terminal Mcs or revert to a stem-like state upon migration into the WNT-negative bulge/ORS^up^. Subsequently, most McSCs home back to the HG. Orange dotted lines in single color images suggest nuclei location. Dashed white lines outline epithelial-dermal boundary. Scale bar: 20 μm, 10 µm in separate channel images. IF: immunofluorescence; HG: hair germ; Bg: bulge; Bb: bulb; ORS^up^: upper ORS; TAM: tamoxifen; d: day; SC: stem cell; TA: transit-amplifying; DF: differentiated. Source data

We next investigated the mechanisms that enable McSC dedifferentiation. Previous studies have shown that constitutive activation of WNT in McSCs leads to their untimely differentiation and eventual loss, whereas inhibition of WNT leads to deficient melanocyte regeneration^7,39,40^. WNT ligands are upregulated in the HG at anagen onset to promote McSC differentiation^7,15,41,42^. As the HG cells grow down to form the HF bulb, the WNT active area also moves down as the anagen phase progresses (Extended Data Fig. 11a). Nonetheless, our live imaging demonstrated that HG McSCs can translocate to the anagen bulge/ORS^up^ (Figs. 1c and 3e and Extended Data Fig. 2b), where WNT signalling is known to be suppressed. We proposed that downregulation of WNT ligands following migration to the bulge/ORS^up^ may permit the dedifferentiation of McSCs.

First, to understand how WNT status correlates with differentiation status, we examined scRNA-seq data. The analysis showed that WNT activation genes were upregulated during early anagen, especially in late-stage *Oca2*^+^ McSCs (Extended Data Fig. 11b,c). Immunofluorescence data confirmed that *Oca2*^+^ McSCs activate WNT signalling in anagen onset HG (Extended Data Fig. 11d–f). Notably, WNT signalling was subsequently downregulated by mid/late anagen in the bulge/ORS^up^ regardless of UVB irradiation (Extended Data Fig. 11d–f).

**Extended Data Fig. 12:**
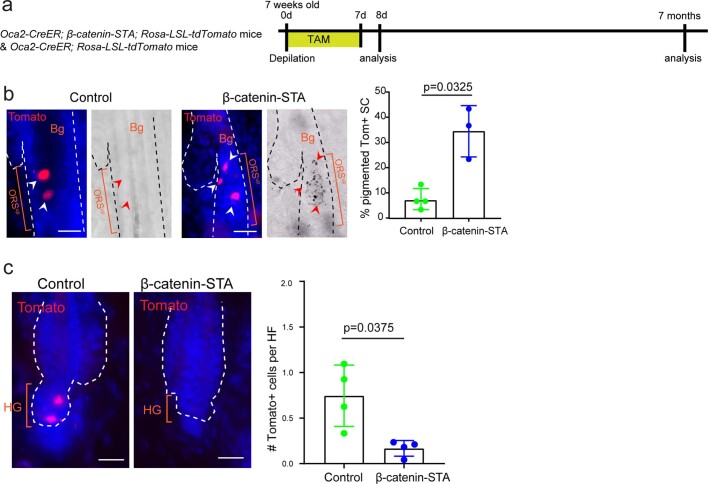
Constitutive WNT activation induces pigmentation of Oca2^+^ McSCs, leading to their loss. (**a**) Experimental scheme: *Oca2-CreER;Rosa-LSL-tdTomato* control and *Oca2-CreER;β-catenin-STA;Rosa-LSL-tdTomato* mice were depilated and injected with TAM for 7 days. (**b**) Evaluation of pigmentation status of Tom^+^ McSCs with or without constitutive WNT activation. Z-stack images of Tom^+^ cells and corresponding brightfield images at 1 day after last TAM injection. Percentages of pigmented Tom^+^ McSCs. Data are represented as mean ± SD. P-values by two-tailed unpaired t test. N = 4 mice (control), N = 2 mice, 3 independent areas (β-catenin-STA). 30 HFs analyzed per mouse/area. (**c**) Evaluation of how long-term constitutive WNT activation affects the maintenance of Oca2^+^ McSCs. Z-stack images of Tom^+^ cells at 7 months post-TAM injection. Number of Tom^+^ McSCs per hair follicle in the telogen bulge/HG. Data are represented as mean ± SD. P-value by two-tailed unpaired t test. N = 4 mice in control, N = 4 independent areas from 2 mice in β-catenin-STA group. At least 15 HFs analyzed per mouse/area. Dashed lines outline epithelial-dermal boundary. Arrowheads point to Tom^+^ McSCs in the bulge/ORS^up^. Scale bar: 20 µm. HG: hair germ; Bg: bulge; ORS^up^: upper ORS; TAM: tamoxifen; d: day. Source data

Second, we verified whether persistent WNT activation in anagen *Oca2*^+^ McSCs precludes reversion to an undifferentiated McSC state. Constitutive activation of WNT signalling by expressing stabilized β-catenin (encoded by *Ctnnb1*) in *Oca2*^+^ McSCs (from *Oca2*^*creER*^*;Ctnnb1*^*STA*^*;Rosa*^*LSL-tdTomato*^ mice^43^) showed that in this environment, these cells survived and inappropriately continued along the differentiation pathway to express pigment after translocation into the anagen bulge/ORS^up ^(Extended Data Fig. 12a,b). By contrast, normal *Oca2*^+^ cells reverted to an undifferentiated state (Extended Data Fig. 12a,b). Thus, downregulation of WNT activity permits the dedifferentiation of *Oca2*^+^ McSCs. When followed up long term, tdTomato^+^ McSCs in the β-catenin stabilized mice showed a significant reduction (Extended Data Fig. 12c), a result consistent with previous work demonstrating that long-term WNT activation depletes McSCs^7^.

**Fig. 4:**
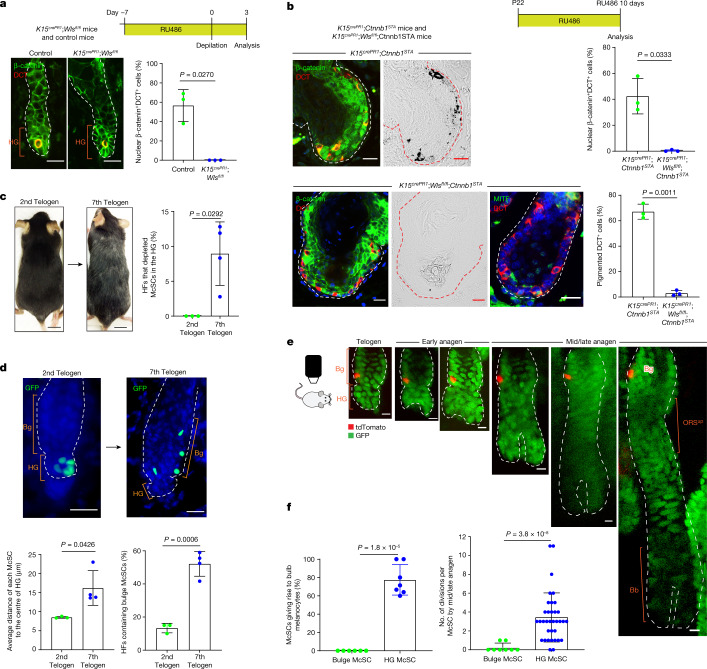
HF ageing limits the access of McSCs to niche signals that reversibly regulate their differentiation. **a**, Top, *K15*^*crePR1*^*;Wls*^*fl/fl*^ conditional knockout mice and control mice (7 weeks old) were treated with RU486 and depilated. Bottom left, immunofluorescence of DCT and β-catenin at 3 days after depilation. Bottom right, percentages of DCT^+^ McSCs with nuclear β-catenin. *N* = 3 mice. **b**, Top, *K15*^*crePR1*^*;Ctnnb1*^*STA*^ mice and *K15*^*crePR1*^*;Wls*^*fl/fl*^*;Ctnnb1*^*STA*^ mice were treated with RU486 from P22. Bottom left, immunofluorescence of DCT, β–catenin and MITF with corresponding bright-field images. Bottom right, percentages of DCT^+^ McSCs with nuclear β-catenin signals and pigmentation. *N* = 3 mice. **c**,**d**, Comparison of *Dct*^*rtTA*^*;tetO*^*H2B-GFP*^ mice at second and seventh telogen. **c**, Representative images of mice. Percentages of HFs lacking HG McSC (second telogen: *N* = 3 mice, 20 HFs analysed per mouse. Seventh telogen: *N* = 4 mice, ≥36 HFs analysed per mouse). **d**, Left, *z*-stack images of HFs with McSCs in GFP. Right, average distance of each McSC from the HG centre (second telogen, *N* = 3 mice; seventh telogen, *N* = 4 mice. Five HFs analysed per mouse). Right, percentages of HFs containing bulge McSCs (second telogen, *N* = 3 mice, 20 HFs analysed per mouse; seventh telogen, *N* = 4 mice, ≥36 HFs analysed per mouse). **e**, Live tracing of a single tdTomato^+^ bulge McSC in a *Tyr*^*creER*^*;Rosa*^*LSL-tdTomato*^*;K14*^*rtTA*^*;tetO*^*H2B-GFP*^ mouse. **f**, Left, percentages of bulge and HG McSCs that produced differentiated progeny (HG, *N* =  7 mice, 59 HFs; bulge, *N* = 6 mice, 8 HFs). Right, the number of divisions by each bulge and HG McSC by mid/late anagen (*N* = 35 HFs with a single HG McSC; 8 HFs with a single bulge McSC). For **a**–**d**,**f**, data are presented as the mean ± s.d. *P* values derived by two-tailed unpaired *t*-test. Dashed white or red lines outline the epithelial–dermal boundary (**a**,**b**,**d**,**e**). Scale bars, 20 µm (**a**,**b**,**d**), 10 µm (**e**) or 1 cm (**c**). Source data

Last, to confirm that WNT active status is regulated by epithelial niche cells, we used *K15*^*crePR1*^*;Wls*^*fl/fl*^ mice to inducibly delete Wntless (*Wls*) and prevent WNT ligand release specifically from K15^+^ epithelial cells that surround McSCs in the bulge and HG^7,44–46^. Following induction with RU486 and subsequent anagen onset, these mice exhibited defects in nuclear β-catenin expression in McSCs and in surrounding epithelial cells (Fig. 4a). This result suggested that epithelial-derived WNT ligands have an essential role in McSC WNT activation. To ensure that only McSCs were disrupted in WNT activity, we also examined McSCs in RU486-treated *K15*^*crePR1*^*;Wls*^*fl/fl*^*;Ctnnb1*^*STA*^ mice^43^, in which *Wls* is deleted but β-catenin signalling is retained in epithelial cells. Previously published results^7^ and Fig. 4b show that constitutive WNT activation in control RU486-treated *K15*^*crePR1*^*;Ctnnb1*^*STA*^ HFSCs resulted in aberrant but pigmented HF development. However, in mice lacking *Wls*, epithelial niche cells showed WNT activation and nuclear β-catenin localization, despite their inability to secrete WNT ligands (Fig. 4b). By contrast, DCT^+^ melanocytes did not exhibit nuclear β-catenin, they did not express the melanocyte differentiation marker MITF or produce pigment (Fig. 4b). These results demonstrate the requirement of epithelial WNT ligands for McSC WNT activation. Together, these findings show that niche-derived WNT ligands regulate the reversible differentiation of McSCs.

## Repetitive plucking increases dormant bulge McSCs

The above results suggest that the ability of McSCs to translocate between the stem cell (WNT^–^ bulge/ORS^up^) and TA (WNT^+^ HG/bulb) compartments of the HF may enable them to reversibly attain distinct differentiation states. However, our live imaging showed that not all McSCs originating from the HG home back to the HG compartment at the next telogen phase but rather remain in the telogen bulge (Figs. 1c,g and 3e and Extended Data Fig. 7j). Given the high prevalence of hair greying in humans and mice, we investigated whether this shift in distribution of HG McSCs might correlate with ageing.

**Extended Data Fig. 13:**
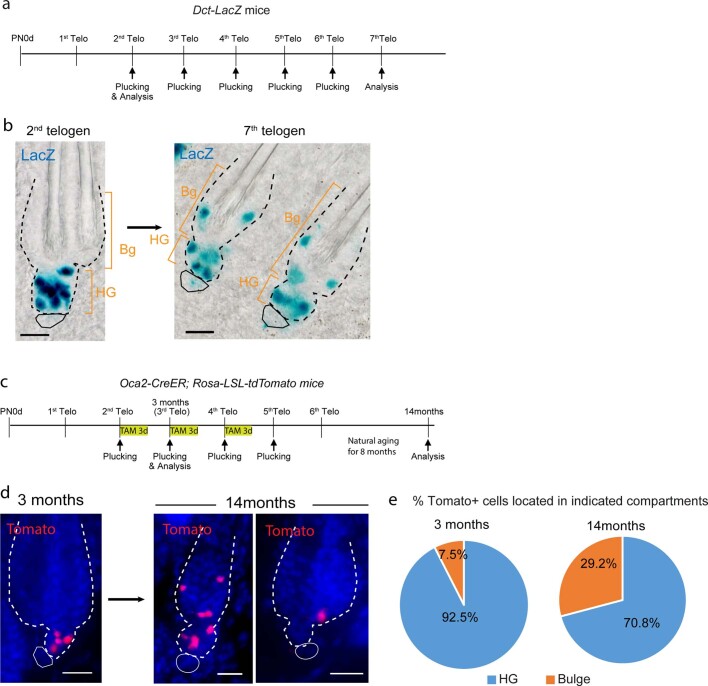
Increased scattering of McSCs to the bulge in aged *Dct-LacZ* and *Oca2-CreER;Rosa-LSL-tdTomato* mice. (**a**) Experimental scheme of repeated plucking of *Dct-LacZ* mice. (**b**) Images of hair follicles from *Dct-LacZ* mice at 2^nd^ telogen and 7^th^ telogen after hair plucking. (**c**) Experimental scheme: early repeated plucking followed by natural aging of *Oca2-CreER;Rosa-LSL-tdTomato* mice. (**d**) Z-stack images of hair follicles from *Oca2-CreER;Rosa-LSL-tdTomato* mice at 3 months old (3rd telogen) and 14 months old telogen. (**e**) Quantification of Tom^+^ McSCs residing in the HG or bulge in *Oca2-CreER;Rosa-LSL-tdTomato* mice at indicated time points. N = 42 HFs from 2 mice at 3 months. N = 54 HFs from 2 mice at 14 months. Dashed lines outline epithelial-dermal boundary. Solid circles suggest location of dermal papilla. Scale bar: 20 µm. HG: hair germ; Bg: bulge; TAM: tamoxifen; d: day; PN: postnatal; Telo: telogen. Source data

To this end, we experimentally accelerated the ageing of HFs by repeated depilation of *Dct*^*LacZ*^ mice and *Dct*^*rtTA*^*;tetO*^*H2B-GFP*^ mice at every telogen phase starting from postnatal day 21 (P21)^17,18,47^. Consistent with previous studies^1^, we detected HFs with significant loss of McSCs, which was evident in the HG by the seventh telogen phase (Fig. 4c). As expected, these mice displayed hair greying (Fig. 4c). Notably, many McSCs in aged HFs had changed location and were scattered to the bulge rather than being tightly aggregated within the HG compartment (Fig. 4d and Extended Data Fig. 13a,b). Image analyses revealed that the distance between individual McSCs was greater in aged HFs than in young HFs (Fig. 4d). The percentage of HFs containing bulge McSCs increased from 10% to more than 50% (Fig. 4d). A similar observation was noted when we examined *Oca2*^*creER*^*;Rosa*^*LSL-tdTomato*^ mice following repeated plucking (≥6 hair cycles) (Extended Data Fig. 13c–e).

To understand whether such abnormal McSC distribution can affect the regeneration of mature melanocytes, we specifically traced the rare bulge McSCs in young telogen HFs through live imaging and assessed their ability to generate differentiated melanocytes. Telogen bulge McSCs gave rise only to bulge/ORS^up^ McSC progeny, without contributing to the differentiated melanocyte compartment (Fig. 4e,f). Moreover, bulge McSCs either remained quiescent or underwent limited division to give rise to McSC progeny (Fig. 4e,f). This was in contrast to HG McSCs, which actively proliferated to regenerate progeny in both stem cell and differentiated cell compartments (Figs. 1c,d and 4f). These results reveal that telogen bulge McSCs, which do not directly contribute to bulb melanocyte regeneration, increase during HF ageing induced by repetitive hair regeneration.

## Discussion

Collectively, our data demonstrate that the McSC system is a thin-layered system in which HG McSCs act as both stem cells and TA cells at the onset of regeneration. The findings reveal a new model whereby dedifferentiation plays an integral part in homeostatic stem cell maintenance (Extended Data Fig. 11f).

This new model of McSC maintenance highlights a previously unknown level of plasticity. We also identified the vulnerability of this system compared with most other stem cell systems that operate through multilayered distinct stem cell and progenitor cell populations with separate functions and locations. McSCs need to be mobile to demonstrate their chameleon-like features, whereby they display a TA phenotype when located in the TA compartment of the HF (the growing HG area) and then a stem cell phenotype once translocated into the stem cell compartment of the HF (Extended Data Fig. 11f). Therefore, HFSC and McSC organization during tissue regeneration may appear to be parallel when stem cell and TA cells are defined by their differentiation phenotype. The crucial difference between these two stem cell systems is that McSCs, which exhibit the TA phenotype in regard to differentiation status and location, can move back and dedifferentiate into stem cells. However, we also demonstrated that McSC movement between the stem cell and TA compartments is not precisely recapitulated during repeated regenerations, resulting in the accumulation of McSCs that fail to undergo this movement during HF ageing.

Previous studies have demonstrated that positions of individual adult stem cells within the niche are unfixed or interchangeable in the HFSC and intestinal systems^12,26,48^. Because McSCs regenerate hair melanocytes only when they are within the HG compartment, their proper localization would be required to prevent hair greying. Current theory proposes that McSCs accumulate genotoxic damage during ageing and are gradually eliminated over time through terminal differentiation in response to anagen niche signals^49^. As such, pro-differentiation signals are likely to be concentrated in the HG compartment^7^, and bulge McSCs, when they exist, may have a higher survival rate owing to reduced exposure to such differentiation stimuli. Under extraordinary conditions, bulge McSCs might act as a valid source of melanocyte production for hair re-pigmentation, but relocation closer to the HG would be a prerequisite for this.

Last, tumours derived from melanocytes (melanomas) retain self-renewal ability regardless of their fully differentiated, pigmented phenotype^50,51^, which is unlike many other tumours, including epithelial cancers. Because of this difference, melanomas are difficult to eliminate and represent the most dangerous form of skin cancer. This study has provided evidence for the plasticity of normal McSCs under physiological conditions. An implication of this finding is that this marked capacity of normal melanocytes to retain self-renewal ability after undergoing differentiation could at least partially underlie the plastic nature of melanoma.

## Methods

### Generation of *Oca2*^*creER*^ knock-in mice

*Oca2*^*creER*^ knock-in line was generated following well-established protocols with slight modifications^52,53^. The *Oca2*^*creER*^-targeting DNA was constructed as presented in Extended Data Fig. 7a by VectorBuilder. The purified plasmid DNA of the targeting vector was linearized using the restriction enzymes NotI and SalI for embryonic stem (ES) cell targeting. The mouse MK6 (C57BL/6J) ES cells (established at New York University (NYU) Langone’s Rodent Genetic Engineering Laboratory) were grown, at passage 6, on mitotically inactivated mouse embryonic fibroblast (MEF) cells (Sigma Millipore) and passaged the day before electroporation. Linearized targeting vector DNA (25 μg ml^–1^) containing the neomycin-resistance gene was added to the cell suspension, and electroporation was performed using either a Gene Pulser II or a Gene Pulser system (Bio-Rad). Following electroporation, the cells were plated onto neomycin-resistant MEFs and incubated at 37 °C, 95% humidity and 5% CO_2_. After 24 h, geneticin (160 μg ml^–1^ active concentration, Invitrogen) was added to the growth medium for positive selection of antibiotic-resistant ES cell colonies. The medium was changed every day, and geneticin (G418) selection was maintained for 6 days. Antibiotic-resistant ES cell colonies were counted, picked and split to grow in 96-well plates duplicated for cryopreservation, and homologous recombination events were identified through genotyping by Southern blot analysis. The mouse MK6 ES cells and MEFs were tested for mycoplasma contamination before use and were not authenticated.

ES cells with successful homologous recombination were injected into mouse blastocyst embryos. ES cells were trypsinized to obtain a single-cell suspension, and the cell suspension was kept on ice in 1 ml ES cell medium in a 15 ml tube until use. Blastocyst embryos were collected from C57BL/6-albino females (4 weeks old, NIH 562, CRL) at 3.5 days post coitum. Ten to fifteen ES cells were injected into each blastocyst embryo, and injected blastocysts were cultured in KSOM medium at 37 °C in an atmosphere of 5% CO_2_ for 2 h until the blastocyst cavity was recovered. The microinjected blastocysts were transferred to the uterine horns of 2.5 days post coitum pseudopregnant females (CD-1, CRL) using the standard procedure to generate chimeric mice. The chimeric mice were bred with C57BL/6 to obtain *Oca2*^*creERT2*^ mice.

### Mouse experiments

All animal experiments were performed in compliance with all relevant ethical regulations for animal testing and research and in accordance with animal protocols approved by the Institutional Animal Care and Use Committee at NYU School of Medicine. Mice were housed in an animal room with a temperature range of 20–22 °C, humidity range of 30–70% and under a 12–12 h dark–light cycle.

*Tyr*^*creER*^ (012328), *Rosa*^*LSL-tdTomato*^ (007905), *Wnt1*^*cre*^, *K15*^*crePR1*^ (005249), *Wls*^*fl/fl*^ (012888) and *K14*^*rtTA*^ (008099) mice were purchased from The Jackson Laboratory. *Dct*^*rtTA*^*;tetO*^*H2B-GFP*^ (iDCT-GFP) mice were obtained from the NCI Mouse Repository. *Dct*^*lacZ*^ mice were from P. Overbeek. *Ctnnb1*^*fl(ex3)*^*/+ (Ctnnb1*^*STA*^) mice were from M. M. Taketo^43^. Mice were bred and crossed in-house to obtain experimental and control animals in mixed backgrounds. Mice from experimental and control groups were randomly selected from either sex for experiments. Data collection and analyses were not performed blind to the conditions of the experiments.

To induce Cre recombination, tamoxifen (Sigma-Aldrich) treatment was performed as previously published^7^ by intraperitoneal injection (0.1 mg g^–1^ body weight) of a 20 mg ml^–1^ solution in corn oil per day. For the UVB experiment, dorsal fur of 3-week-old *Oca2*^*creER*^*;Rosa*^*LSL-tdTomato*^ mice was clipped, and mice were anaesthetized. Mice were treated every other day with 600 mJ cm^–2^ UVB per day 3 times in total. Skin biopsies were taken from euthanized mice or under isoflurane anaesthesia. For isolation of McSCs and differentiated bulb MCs, *Dct*^*rtTA*^*;tetO*^*H2B-GFP*^ mice were administered doxycycline-containing chow (1 g kg^–1^, Bio-Serv) for 4 days before euthanizing the mice for cell isolation. In the c-Kit antibody injection experiment, mice were subcutaneously injected with 150 µl of 0.5 mg ml^–1^ c-Kit antibody (ACK45, BD Pharmingen) into a 2 × 2cm area of back skin. Control mice were subcutaneously injected with 150 µl of PBS.

### Melanocyte stem cell in vivo imaging

In vivo imaging of melanocyte stem cells was based on previously described methods of live imaging of HF stem cells^54,55^. *Tyr*^*creER*^*;Rosa*^*LSL-tdTomato*^*;K14*^*rtTA*^*;tetO*^*H2B-GFP*^ mice were given a single injection of 60 µg tamoxifen on P21 and maintained on a 1 g kg^–1^ doxycycline-containing diet from P21. All the in vivo imaging was performed at least 3 days after tamoxifen induction. As the timing of the hair cycle is slightly variable between individual mice and individual HFs, we kept imaging the mouse to monitor the hair cycle stages. HF stages were determined according to a previous publication^56^ by observing the pattern of GFP-labelled HF epithelial cells. We performed the initial imaging at telogen/anagen I to capture HFs that contain only a single tdTomato-labelled cell. To trace the fate of HG melanocytes, we focused on HFs containing a single tdTomato^+^ melanocyte in the HG and revisited the same HFs every 1–4 days to capture hair cycle stages, including early anagen, mid/late anagen, early-to-mid (early/mid) catagen, late catagen and the subsequent (second) telogen. Some mice were revisited less frequently to focus on capturing mid/late anagen and the subsequent telogen. To trace the fate of bulge McSCs, we initially visited HFs containing a single tdTomato^+^ McSC in the bulge at telogen and revisited the same HFs until mid/late anagen.

To trace the fate of melanocytes located in the TA compartment of early-anagen HFs, we placed *Oca2*^*creER*^*;Rosa*^*LSL-tdTomato*^*;K14*^*ktTA*^*;tetO*^*H2B-GFP*^ mice on a doxycycline-containing diet from P21 and kept imaging the mouse to monitor the hair cycle stages. The mice were given a single injection of 1.2 mg tamoxifen when the HF stage progressed to anagen I/early-anagen II. We then performed the initial imaging at anagen II/anagen IIIa (at least 3 days after tamoxifen injection) to capture HFs that contained only a single tdTomato-labelled cell, which is located in the lowermost part of the HG. The same HFs were revisited at mid/late anagen and telogen.

Throughout the course of imaging, mice were on a warming pad and anaesthetized with vapourized isoflurane delivered through a nose cone (1.5% in oxygen and air). The ear was immobilized on a custom-made stage, and a glass coverslip was placed directly against it. An Olympus Fluoview multiphoton microscope (FVMPE-RS) equipped with a MaiTai HP DS-OL laser tuned to 940 nm and an Insight X3-OL tuned to 1,120 nm (Newport Spectraphysics) were used in line sequential mode to acquire *z*-stacks with a ×25 NA.

### Revisitation of the same HFs in live imaging

To ensure successful revisitation of the same HFs, patterns of HF clusters and blood vessel locations were used as landmarks. Blood vessel location in the ear was first used to broadly return to the same area. Then HF cluster patterns in large areas were recorded using multiple tiled images (up to 30) with *z*-stack steps of 20–30 µm during the initial visit and revisits. Rather than being evenly distributed, HFs in the imaged areas showed distinct clustered patterns. A cluster usually contained three to ten HFs with a gap between different clusters. By numbering the clusters and finding the same clusters over time using a tiled map, we frequently revisited the same cluster. Owing to the low tamoxifen concentration used to achieve clonal labelling of McSCs, only a subset of HFs within a cluster contained any tdTomato-labelled cells. We verified that within a cluster, the number and pattern of HFs that contained tdTomato-labelled cells matched previous visits to a specific HF.

### X-gal staining

X-gal staining was done as previously published^38^. Dorsal skin from *Dct*^*lacZ*^ mice was collected, and subcutaneous fat was removed using scalpel blades (Miltex). Tissues were fixed in 4% paraformaldehyde (PFA) for 30 min at 4 °C, and whole-mount X-gal staining was performed. After X-gal staining, skin samples were fixed again in 4% PFA at 4 °C overnight. The tissues were then subjected to 3D whole-mount analyses.

### 3D whole-mount niche analyses

3D whole-mount niche analysis was done as previously published^38^. Whole skin samples of *Dct*^*LacZ*^ mice were stained with X-gal and sequentially treated with 25% glycerol–PBS, 50% glycerol–PBS and 100% glycerol for 3 h at room temperature or overnight at 4 °C. Skin was then cut into thin strips using a scalpel blade. Single HFs were then dissected and isolated using a dissection microscope (Axiovision Discovery V12).

Skin samples of *Oca2*^*creER*^*;Rosa*^*LSL-Tomato*^ mice, *Tyr*^*creER*^*;Rosa*^*LSL-Tomato*^ mice and *Dct*^*rtTA*^*;tetO*^*H2B-GFP*^ mice were collected and fixed in 4% PFA for 30 min at room temperature to preserve tdTomato signals. Tissues were then incubated in 30% sucrose at 4 °C overnight and embedded in OCT compound (Sakura). Then 70–100-µM-thick skin sections, including whole-mount HFs, were made and counterstained with 4′,6-diamidine-2′-phenylindole dihydrochloride (DAPI).

Whole HFs were then imaged as described in the section ‘Microscopy’.

### Immunofluorescence

Immunofluorescence was done as previously published but with slight modifications^7,24^. Skin tissues were fixed overnight in 4% PFA at 4 °C. After sequential dehydration in increasing concentrations of ethanol and xylene, tissues were embedded in paraffin. Sections were cut at 6 μm, deparaffinized and microwaved in 10 mM Tris and 1 mM EDTA (pH 8.0) for antigen retrieval. Tissue sections were then incubated with the following primary antibodies for 2 h at room temperature or overnight at 4 °C in PBT (PBS plus 0.1% Triton-X100) with 10% FBS, followed by Alexa-488-conjugated or Alexa-594-conjugated secondary antibodies (1:200; Thermo Fisher): goat anti-Dct (1:100; Santa Cruz, sc-10451), rabbit anti-Tomato (1:1,000; Rockland, 600–401–379); mouse anti-Tomato (RF5R) (1:500; Thermo Fisher, MA5–15257); rabbit anti-Tyrp1 (1:100; Sigma-Aldrich, SAB2102617); mouse anti-E-cadherin (1:100; BD Transduction, 610181); rabbit anti-Ki67 (1:100; Abcam, ab15580); mouse anti-β-catenin (1:400; Sigma-Aldrich, C7207); and mouse anti-MITF (1:100; Abcam, ab12039). The following secondary antibodies from Thermo Fisher were used: Alexa Fluor 594 donkey anti-mouse IgG (1:200; A21203); Alexa Fluor 488 donkey anti-mouse IgG (1:200; A21202); Alexa Fluor 594 donkey anti-rabbit IgG (1:200; A21207); Alexa Fluor 488 donkey anti-rabbit IgG (1:200; A21206); Alexa Fluor 594 donkey anti-goat IgG (1:200; 11058); and Alexa Fluor 488 donkey anti-goat IgG (1:200; A11055). Skin sections were counterstained with DAPI.

For detection of CD34, P-cadherin and DCT, skin tissues were collected, processed and embedded in OCT as described above (‘3D Whole mount niche analyses’) to preserve tdTomato or GFP signals. Next, 50–70-µM-thick skin sections were made. The skin sections were incubated in PBS plus 0.5% Triton-X100 for 1 h at room temperature and incubated with primary antibody against DCT (1:100; Santa Cruz, sc-10451), CD34 (1:50; BD Pharmingen, 553731) and P-cadherin (1:100; Invitrogen, 13-2000Z) overnight at 4 °C. Skin sections were then washed 3 times with PBS for 10 min at room temperature. For DCT, sections were incubated with Alexa-488-conjugated secondary antibody (1:200) for 1 h at room temperature. For CD34 and P-cadherin, sections were incubated with biotinylated anti-rat IgG (1:100; Vector Laboratories, BA-9400) for 1 h followed by incubation with streptavidin Alexa 647 conjugate (1:200, Invitrogen, S32357) for 30 min at room temperature. Skin sections were counterstained with DAPI.

### In situ hybridization

RNAscope in situ hybridization was performed using a Leica Bond III automated staining platform (Leica Biosystems) according to the manufacturer’s protocol. Mouse probes of *Oca2* (ACDBio, 1072511), *Gpr143* (ACDBio, 535108), *Col12a1* (ACDBio, 312638) and *Txnip* (ACDBio, 457228) for the Leica System were used, and a RNAscope LS Multiplex Fluorescent assay (ACDBio) was used for detection. The double detection of immunofluorescence was performed after finishing in situ hybridization by applying the primary and secondary antibodies as described in the section ‘Immunofluorescence’. Skin sections were then scanned using Vectra Polaris (Akoya Biosciences) at ×20 for Opal fluors 570 (Akoya Biosciences, FP1488001KT), 690 (Akoya Biosciences, FP1497001KT) and DAPI (Akoya Biosciences, FP1490). Scanned images were processed using Inform (v.2.6.0) software (Aloya Biosciences). For quantitative analysis, HALO (v.3.5) software (module Indica Labs-FISH v.3.2.3) was utilized. FISH probe cell intensity (average intensity of FISH probe (×) spots and clusters per cell) was measured in tdTomato^+^ cells in the mid-anagen bulge/ORS^up^ and the bulb. The relative intensity of bulge/ORS^up^ tdTomato^+^ cells to bulb tdTomato^+^ cells within the same sample was compared across multiple samples.

### Microscopy

For thin sections, images were taken at a single focal plane. For whole-mount tissues and thick sections, serial *z*-images were collected throughout the depth of each entire HF. Wide-field fluorescence images were taken with standard narrow-pass filters with an Eclipse Ti inverted microscope (Nikon) or an upright Axioplan (Zeiss). Images were processed to reconstruct a focused image using ImageJ/Fiji, Adobe Photoshop and the extended depth of focus function in the NIS-Elements software (Nikon, v.5.20.02).

The *z*-stack fluorescent images in Figs. 2a and 3b were taken using a LSM 880 confocal microscope with a ×63 NA/1.4 Plan Apochromat lens (Zeiss). The *z*-stacks were taken at 0.3 µm steps. Volumes were reconstructed using Imaris 9.5 software (Oxford Instruments).

### Single-cell dissociation

Single melanocyte isolation was performed as previously published^24^ but with slight modifications. To isolate a single melanocyte from anagen II HFs, *Dct*^*rtTA*^*;tetO*^*H2B-GFP*^ mice were depilated at 8 weeks old and fed a doxycycline-containing diet (1 g kg^–1^) for 4 days. At 4 days after depilation, when the HFs are at anagen II, mice were killed. The mice were then rinsed in betadine followed by 70% ethanol. The back skin of mice was collected. Scalpel blades were used to remove subcutaneous fat, and skin was rinsed in PBS and cut into 1 × 1 cm pieces followed by incubation in 0.25% trypsin for 1 h 30 min at 37 °C. Epidermis was separated from the dermis using forceps and scalpel blades, and the epidermis was finely chopped and incubated in 0.25% trypsin for 30 min at 37 °C while shaking at 100 r.p.m., followed by gentle pipetting to obtain a single-cell suspension. The obtained McSC suspension was filtered through a 70 µm nylon filter and centrifuged at 200 r.c.f. for 5 min and resuspended in medium A (DMEM, 10% FBS and 1× penicillin–streptomycin).

To isolate single bulb melanocytes, *Dct*^*rtTA*^*;tetO*^*H2B-GFP*^ were fed a doxycycline-containing diet for 4 days at 5 weeks old, when the HFs are at the anagen VI stage. Isolation of bulb melanocytes was performed according to previously described methods^57^ but with slight modifications. Mice were euthanized and rinsed in betadine followed by 70% ethanol. Skin was cut into 0.5 cm^2^ sections and incubated in 5 mM EDTA (pH 8) and PBS for 2 h at 37 °C. Following incubation, connective tissue, the adipocyte layer and dermis were removed using forceps, and 0.5 cm^2^ skin samples were cut into single rows of HFs and kept in medium A. HF bulbs were microdissected with a surgical blade, collected into medium A and centrifuged for 5 min at 200 r.c.f. Medium A was removed and hair bulbs were incubated in 1 ml of 0.2% collagenase II and 50 U ml^–1^ dispase (9:1 solution) and shaken at 100 r.p.m. for 25 min at 37 °C. Next 400 U ml^–1^ DNase I was added and incubated for 5 min at room temperature. Five volumes of medium A was added and then filtered through a 100 µm cell strainer. Cell suspension was pelleted by centrifuging at 200 r.c.f. for 5 min at 4 °C and resuspended in medium A.

Single GFP^+^ and DAPI-excluded live melanocytes were then isolated by cell sorting on a Sony SY3200 cell sorter equipped with a WinList 3D Analyzer (v.8.0) with a 100 µm nozzle. Single-cell suspensions from four mice of each condition were combined for subsequent scRNA-seq analyses. FlowJo 10.8.2 (Mac only) was used to plot the FACS gating strategy.

### scRNA-seq and data analysis

Single melanocyte suspensions were loaded on a 10x Genomics Chromium instrument to generate single-cell gel beads in emulsion (GEMs). Approximately 5,000–10,000 cells were loaded per channel. The scRNA-seq library for differentiated bulb melanocytes was prepared using the following Chromium Single Cell 3′ v2 reagent kits: Chromium Single Cell 3′ Library & Gel Bead kit v2 PN-120237; Single Cell 3′ Chip kit v2 PN-120236; and i7 Multiplex kit PN-120262 (10x Genomics). The Single Cell 3′ Reagent kits v2 User Guide (Manual Part CG00052 RevA) was followed^58^. The scRNA-seq library for anagen II melanocytes was prepared using the following Chromium Single Cell 3′ v3 reagent kits: Chromium Single Cell 3′ Library & Gel Bead kit v3 PN-1000075; Single Cell 3ʹ Feature Barcode Library kit PN-1000079; Single Cell B Chip Kit PN-1000073; and i7 Multiplex Kit PN-120262 (10x Genomics). The Single Cell 3′ Reagent kits v3 User Guide (Manual Part CG000201 RevA) was followed. Libraries were run on an Illumina NovaSeq 6000. The Cell Ranger Single-Cell software suite (v.6.0.1) was used to perform sample de-multiplexing, barcode processing and single-cell 3′ gene counting. The cDNA insert was aligned to the mm10/GRCm38 reference genome (mm10-2020-A). The undifferentiated telogen melanocyte dataset was downloaded from the NCBI Gene Expression Omnibus (identifier GSE113502) and re-processed with the same version of the Cell Ranger Single-Cell Software Suite (v.6.0.1) and mapped to the same version of reference genome (mm10-2020-A). Further analysis and visualization were performed using Seurat package (v.4.1.0)^59^, using R Studio Desktop (v.1.4.1717) and R (v.4.1.2).

The Seurat object for each condition was generated from digital gene expression matrices. In the quality control step, the parameter of subset cells is nFeature_RNA (200–2000) for telogen melanocytes, nCount_RNA>10000 for anagen II melanocytes and nCount_RNA > 5000 for bulb melanocytes and percentage of mitochondria genes < 0.05 for all conditions. Different thresholds in filtering out low-quality cells were set for each condition owing to the differences of sequencing depth among the conditions. Data were then log scaled, centred and normalized to the number of Unique Molecular Identifier (nUMI). Principal components (PCs) were calculated using Seurat’s RunPCA function. The top 3,000 variable genes were used for calculating PCs.

For each condition, UMAP dimension reduction was performed on the normalized, centred, scaled nUMI count matrices using the first ten PCs. We then performed unsupervised clustering using the Seurat SNN clustering package, using a resolution of 0.6. Most clusters were identified as melanocytes based on expression for *Dct*, whereas each condition contained a minor cluster of epidermal cells positive for *Krt10* or *Krt14*. The epidermal cells were excluded from subsequent analysis.

For comparative analysis, we merged melanocyte matrices from three conditions (telogen melanocyte, anagen II melanocyte and differentiated melanocyte) followed by a standard workflow of Seurat. Data were log normalized, scaled and centred after regressing out the effect of nCount_RNA (the total number of molecules detected within a cell from sequencing) and percent.mt (mitochondria ratio, defined by the PercentageFeatureSet function in Seurat). As the three conditions showed differences in sequencing depth (nCount_RNA varies greatly) and sequencing depth is a major cause of batch effects in scRNA-seq, the effect of nCount_RNA was regressed out before identifying PCs to account for such batch effects. The top 3,000 variable genes were used for calculating PCs. UMAP dimension reduction was performed on the normalized, centred, scaled nUMI count matrices using the first three PCs.

To estimate the cell cycle stage of a cell, we used Seurat’s cell cycle scoring. In brief, averaged relative expression of cell cycle related genes were used to calculate G2/M and S scores, which were used for binning cells into G2/M, S and G1/G0 bins. The cell cycle scores were regressed out in ScaleData step in the analysis of the anagen II melanocyte dataset alone, followed by PC calculation, UMAP dimension reduction and unbiased clustering as described above.

Pseudotime analysis was performed using the slingshot package (v.2.2.0)^60^. The Seurat object was imported into slingshot using the as.SingleCellExperiment function. Then a pseudotime trajectory was constructed using the slingshot function with UMAP dimensional reduction.

### Gene set enrichment analysis

Genes differentially expressed between anagen II melanocytes compared with telogen melanocytes (*P* < 0.05, log_2_(fold change) > 0.25) were rank-ordered from high to low on the basis of their fold change. The pre-ranked gene list as queried for its enrichment in two annotated gene sets acquired from The Molecular Signature Database (MSigDB)—GOBP_DENDRITE_DEVELOPMENT and GOBP_DENDRITE_MORPHOGENESIS—using the preranked gene set enrichment analysis (GSEA) analysis tool^61,62^. A false discover rate *q*-value of <0.25 was deemed significant.

### Quantification and statistical analyses

The measurement of quantifications can be found in the *y* axes of bar plots in the figure and in figure legends. The statistical details of each plot can be found in the figure (*N* number). The exact meaning of *N* is described in the corresponding figure legend. No statistical methods were used to predetermine sample sizes, but our sample sizes were similar to those reported in previous publications^7,24,38–40,55^. Pairwise comparisons between two groups were performed using two-tailed unpaired *t*-test. Comparisons of multiple groups were performed using one-way analysis of variance (ANOVA) or two-way ANOVA followed by multiple comparison test. Details of the statistical test are specified in the figure legends. Statistical significances were considered significant if *P* < 0.05. Exact *P* values are indicated in the figures and legends. Experimental data are shown as the mean ± standard deviation or mean ± standard error of the mean. Statistical analyses and plotting were done using GraphPad Prism (v.9.2.0) and Microsoft Excel (v.2016).

### Material availability

Materials generated in this study can be provided upon reasonable requests by contacting the corresponding author.

### Reporting summary

Further information on research design is available in the Nature Portfolio Reporting Summary linked to this article.

## Online content

Any methods, additional references, Nature Portfolio reporting summaries, source data, extended data, supplementary information, acknowledgements, peer review information; details of author contributions and competing interests; and statements of data and code availability are available at 10.1038/s41586-023-05960-6.

## Supplementary information

Reporting Summary

## Data Availability

All RNA-seq data reported in this paper are deposited into the NCBI Gene Expression Omnibus database. The accession number is GSE203051. The telogen melanocyte dataset, which was previously deposited, has the identifier GSE113502. The mm10/GRCm38 reference genome (mm10-2020-A) utilized in the scRNA-seq analysis is available at https://support.10xgenomics.com/single-cell-gene-expression/software/release-notes/build#mm10_2020A. The GOBP_DENDRITE_DEVELOPMENT dataset used in the GSEA analysis was downloaded from the MSigDB (http://www.gsea-msigdb.org/gsea/msigdb/index.jsp) at http://www.gsea-msigdb.org/gsea/msigdb/mouse/geneset/GOBP_DENDRITE_DEVELOPMENT.html. The GOBP_DENDRITE_MORPHOGENESIS dataset used in the GSEA analysis was downloaded from the MSigDB at http://www.gsea-msigdb.org/gsea/msigdb/mouse/geneset/GOBP_DENDRITE_MORPHOGENESIS.html. Source data are provided with this paper.

## Source data

Source Data Fig. 1
Source Data Fig. 2
Source Data Fig. 3
Source Data Fig. 4
Source Data Extended Data Fig. 1
Source Data Extended Data Fig. 4
Source Data Extended Data Fig. 5
Source Data Extended Data Fig. 7
Source Data Extended Data Fig. 9
Source Data Extended Data Fig. 10
Source Data Extended Data Fig. 11
Source Data Extended Data Fig. 12
Source Data Extended Data Fig. 13

## Code availability

All the bioinformatics analyses were performed using the following previously published packages with publicly available codes: Cell Ranger Single-Cell software (https://support.10xgenomics.com/single-cell-gene-expression/software/pipelines/latest/what-is-cell-ranger); Seurat (https://satijalab.org/seurat/); slingshot (https://bioconductor.org/packages/devel/bioc/vignettes/slingshot/inst/doc/vignette.html); and GSEA Broad Institute (https://software.broadinstitute.org/gsea/index.jsp).
