# Supplementary material for: Dedifferentiation maintains melanocyte stem cells in a dynamic niche
Source: Nature. 2023 Apr 19;616(7958):774–82. doi: 10.1038/s41586-023-05960-6 (PMC10132989; doi:10.1038/s41586-023-05960-6)
Supplement: Supplementary file 1 — Reporting Summary [file 41586_2023_5960_MOESM1_ESM.pdf]

nature portfolio

Corresponding author(s): Mayumi Ito

Last updated by author(s): 02/28/2023

Reporting Summary

Nature Portfolio wishes to improve the reproducibility of the work that we publish. This form provides structure for consistency and transparency in reporting. For further information on Nature Portfolio policies, see our Editorial Policies and the Editorial Policy Checklist.

Statistics

For all statistical analyses, confirm that the following items are present in the figure legend, table legend, main text, or Methods section.

n/a	Confirmed	
☐	☒	The exact sample size (*n*) for each experimental group/condition, given as a discrete number and unit of measurement
☐	☒	A statement on whether measurements were taken from distinct samples or whether the same sample was measured repeatedly
☐	☒	The statistical test(s) used AND whether they are one- or two-sided<br>*Only common tests should be described solely by name; describe more complex techniques in the Methods section.*
☒	☐	A description of all covariates tested
☒	☐	A description of any assumptions or corrections, such as tests of normality and adjustment for multiple comparisons
☐	☒	A full description of the statistical parameters including central tendency (e.g. means) or other basic estimates (e.g. regression coefficient) AND variation (e.g. standard deviation) or associated estimates of uncertainty (e.g. confidence intervals)
☐	☒	For null hypothesis testing, the test statistic (e.g. *F*, *t*, *r*) with confidence intervals, effect sizes, degrees of freedom and *P* value noted<br>*Give P values as exact values whenever suitable.*
☒	☐	For Bayesian analysis, information on the choice of priors and Markov chain Monte Carlo settings
☒	☐	For hierarchical and complex designs, identification of the appropriate level for tests and full reporting of outcomes
☒	☐	Estimates of effect sizes (e.g. Cohen's *d*, Pearson's *r*), indicating how they were calculated

Our web collection on statistics for biologists contains articles on many of the points above.

Software and code

Policy information about availability of computer code

Data collection	Single cell RNA-seq were acquired with an Illumina NovaSeq 6000.<br>Microscopic images were taken with an Eclipse Ti inverted microscope, an upright Axioplan, a LSM 880 confocal microscope, a Vectra Polaris scanner or an Olympus Fluoview multiphoton microscope (FVMPE-RS) .<br>Cell sorting was performed on a Sony SY3200 cell sorter.
Data analysis	For single cell RNA-seq, the Cell Ranger Single-Cell Software Suite, version 6.0.1, was used to perform sample de-multiplexing, barcode processing, and single-cell 3' gene counting. Further analysis and visualization was performed using Seurat package (version 4.1.0) (https://satijalab.org/seurat/), using R Studio Desktop (version 1.4.1717) and R (version 4.1.2), slingshot package (version 2.2.0).<br>Plots were drawn and statistical tests were performed using Graphpad Prism (version 9.2.0) and Microsoft Excel (2016).<br>In situ hybridization data were processed with Inform (v2.6.0) Software and analyzed with HALO (v3.5) software (module Indica Labs-FISH v3.2.3). FACS sorting used WinList 3D Analyzer (version 8.0). FlowJo 10.8.2 (Mac Only) was used to plot FACS gating strategy. Microscopic images were analyzed with ImageJ/Fiji (v1.53c), Adobe Photoshop (Version 21.2.6 20210302.r.482), NIS-Elements software (Version 5.20.02). Volumes were reconstructed with Imaris 9.5 software (Oxford Instruments).

For manuscripts utilizing custom algorithms or software that are central to the research but not yet described in published literature, software must be made available to editors and reviewers. We strongly encourage code deposition in a community repository (e.g. GitHub). See the Nature Portfolio guidelines for submitting code & software for further information.

Data

Policy information about availability of data

All manuscripts must include a data availability statement. This statement should provide the following information, where applicable:

- Accession codes, unique identifiers, or web links for publicly available datasets
- A description of any restrictions on data availability
- For clinical datasets or third party data, please ensure that the statement adheres to our policy

All RNA-seq data reported in this paper are deposited in NCBI Gene Expression Omnibus (GEO) database. The accession number is GSE203051 (https://www.ncbi.nlm.nih.gov/geo/query/acc.cgi?acc=GSE203051). The telogen Mc dataset was previously deposited at GSE113502 (https://www.ncbi.nlm.nih.gov/geo/query/acc.cgi?acc=GSE113502). mm10/GRCm38 reference genome (mm10-2020-A) utilized in scRNA-seq analysis is available at https://support.10xgenomics.com/single-cell-gene-expression/software/release-notes/build#mm10_2020A. The GOBP_DENDRITE_DEVELOPMENT dataset used in the GSEA analysis was downloaded from the MSigDB (http://www.gsea-msigdb.org/gsea/msigdb/index.jsp) at http://www.gsea-msigdb.org/gsea/msigdb/mouse/geneset/GOBP_DENDRITE_DEVELOPMENT.html. The GOBP_DENDRITE_MORPHOGENESIS dataset used in the GSEA analysis was downloaded from the MSigDB at http://www.gsea-msigdb.org/gsea/msigdb/mouse/geneset/GOBP_DENDRITE_MORPHOGENESIS.html.

Human research participants

Policy information about studies involving human research participants and Sex and Gender in Research.

Reporting on sex and gender	This study does not contain human research participants.
Population characteristics	This study does not contain human research participants.
Recruitment	This study does not contain human research participants.
Ethics oversight	This study does not contain human research participants.

Note that full information on the approval of the study protocol must also be provided in the manuscript.

Field-specific reporting

Please select the one below that is the best fit for your research. If you are not sure, read the appropriate sections before making your selection.

☒ Life sciences ☐ Behavioural & social sciences ☐ Ecological, evolutionary & environmental sciences

For a reference copy of the document with all sections, see nature.com/documents/nr-reporting-summary-flat.pdf

Life sciences study design

All studies must disclose on these points even when the disclosure is negative.

Sample size	No statistical methods were used to pre-determine sample sizes, but our sample sizes are similar to those reported in previous publications (refs 7,24,37,38,39,54 of the manuscript). All experiments were repeated at least three times unless specially noted. For single cell RNA-seq, cells isolated from at least 4 independent animal repeats were combined before sequencing.
Data exclusions	No animals were excluded from the analysis.
Replication	All experiments were repeated at least three times (3 biological independent experiments/mice), except for two experiments noted in their legends, which was performed with 2 independent animals. For single cell RNA-seq, cells isolated from 4 independent animal repeats were combined before sequencing. Detailed N number and the exact meaning of N was explained in the figure legends.
Randomization	The experiments were not randomized because the mice used in this study were selected according to their correct genotype. Sex-specific differences were minimized by including similar number of male and female animals. Age-specific differences were minimized by using mice of comparable age in different experimental groups
Blinding	The investigators were not blinded to allocation during experiments and outcome assessment in the majority of analyses (except for analyses in Extended Data Fig. 12c and Extended Data Fig. 13b). Blinding was not possible as the same investigators processed the animals and analyzed the data.

Reporting for specific materials, systems and methods

We require information from authors about some types of materials, experimental systems and methods used in many studies. Here, indicate whether each material, system or method listed is relevant to your study. If you are not sure if a list item applies to your research, read the appropriate section before selecting a response.

Materials & experimental systems

n/a	Involved in the study
☐	☒ Antibodies
☐	☒ Eukaryotic cell lines
☒	☐ Palaeontology and archaeology
☐	☒ Animals and other organisms
☒	☐ Clinical data
☒	☐ Dual use research of concern

Methods

n/a	Involved in the study
☒	☐ ChIP-seq
☐	☒ Flow cytometry
☒	☐ MRI-based neuroimaging

Antibodies

Antibodies used

Goat anti-Dct (1:100; Santa Cruz #sc-10451), Rabbit anti-Tomato (1:1000; Rockland #600–401–379), Mouse anti-Tomato (RF5R) (1:500; Thermo Fisher #MA5–15257), Rabbit anti-Tyrp1 (1:100; Sigma-Aldrich #SAB2102617), Mouse anti-E-cadherin (1:100, BD Transduction #610181), Rabbit anti-Ki67 (1:100; Abcam #15580), Mouse anti-β-catenin (1:400; Sigma-Aldrich #C7207), Mouse anti-MITF (1:100; Abcam #ab12039), Rat anti-Cd34 (1:50; BD Pharmingen #553731) and Rat anti-p-cadherin (1:100; Invitrogen #13-2000Z). Alexa Fluor 594 donkey anti-mouse IgG (1:200, Thermo Fisher #A21203), Alexa Fluor 488 donkey anti-mouse IgG (1:200, Thermo Fisher #A21202), Alexa Fluor 594 donkey anti-rabbit IgG (1:200, Thermo Fisher #A21207), Alexa Fluor 488 donkey anti-rabbit IgG (1:200, Thermo Fisher #A21206), Alexa Fluor 594 donkey anti-goat IgG (1:200, Thermo Fisher #A11058), Alexa Fluor 488 donkey anti-goat IgG (1:200, Thermo Fisher #A11055). Biotinylated anti-rat IgG (1:100, Vector Laboratories #BA-9400), Alexa 647 conjugate (1:200, Invitrogen #S32357)

Validation

The usage of the below antibodies on mouse skin tissues have been validated by previous literatures (Sun et al., 2019, ref24). goat anti-Dct, Rabbit anti-Tomato, Mouse anti-Tomato, Mouse anti-E-cadherin, Rabbit anti-Ki67, Mouse anti-β-catenin, Mouse anti-MITF.
The below antibodies have been validated by the manufacture for usage with mouse species and for immunohistochemistry:
Rabbit anti-Tyrp1 (https://www.sigmaaldrich.com/US/en/product/sigma/sab2102617)
Rat anti-Cd34 (https://www.bdbiosciences.com/en-us/products/reagents/flow-cytometry-reagents/research-reagents/single-color-antibodies-ruo/purified-rat-anti-mouse-cd34.553731)
Rat anti-p-cadherin (https://www.thermofisher.com/antibody/product/P-cadherin-Antibody-clone-PCD-1-Monoclonal/13-2000Z)

Eukaryotic cell lines

Policy information about cell lines and Sex and Gender in Research

Cell line source(s)	mouse MK6 (C57BL/6J) ES cells: established at NYU Langone's RGEL; murine embryonic fibroblast (MEF) cells: Sigma Millipore
Authentication	None
Mycoplasma contamination	Tested before use.
Commonly misidentified lines (See ICLAC register)	None

Animals and other research organisms

Policy information about studies involving animals; ARRIVE guidelines recommended for reporting animal research, and Sex and Gender in Research

Laboratory animals	Oca2-CreER mice were generated with the Rodent Genetic Engineering Laboratory of NYU Langone. Tyr-CreER (#012328), Rosa-LSL-tdTomato (#007905), Wnt1-Cre, Wntless fl/fl, K15-CrePR1 and K14-rtTA (#008099) mice were purchased from The Jackson Laboratory. Dct-rtTA; tetO-H2B-GFP (iDCT-GFP) mice were obtained from NCI Mouse Repository. Dct-lacZ mice were from Dr. Paul Overbeek. β-catenin fl(ex3)/+mice were from Dr. M. Mark Taketo. C57BL/6-albino females (4 weeks old, NIH 562) were purchased from Charles River Laboratories. All mice utilized in this study are of 3weeks to 2 years of age.
Wild animals	No wild animals were used in this study.
Reporting on sex	Both male and female mice were used in the study. Sex-specific differences were minimized by including similar number of male and female animals.
Field-collected samples	No field-collected samples were used in this study.
Ethics oversight	The Institutional Animal Care and Use Committee (IACUC) at New York University School of Medicine

Note that full information on the approval of the study protocol must also be provided in the manuscript.

Flow Cytometry

Plots

Confirm that:

☒ The axis labels state the marker and fluorochrome used (e.g. CD4-FITC).

☒ The axis scales are clearly visible. Include numbers along axes only for bottom left plot of group (a 'group' is an analysis of identical markers).

☒ All plots are contour plots with outliers or pseudocolor plots.

☒ A numerical value for number of cells or percentage (with statistics) is provided.

Methodology

Sample preparation	Single melanocytes were isolated from the back skin of Dct-rtTA; tetO-H2B-GFP mice. To isolate anagen II melanocytes, mouse skin was incubated in 0.25% Trypsin for 2 hr at 37° C. Epidermis was separated from the dermis using forceps and scalpel blades and the epidermis was chopped finely and stirred at RT for 20 min Media A (DMEM, 10% FBS, 1x penicillin/ streptomycin) to obtain single cell suspension. To isolated bulb melanocytes, hair follicle bulb was micro-dissected with a surgical blade, collected into Media A, and centrifuged for 5 min at 200 rcf. Media A was removed and hair bulbs were incubated in 1 mL of 0.2% collagenase II and 50 U/mL dispase (9:1 solution) and shaken at 100 rpm for 25 minutes at 37° C to obtain single cell suspension.
Instrument	Sony SY3200 cell sorter
Software	WinList 3D Analyzer (version 8.0) was used for collecting data using Sony SY3200 cell sorter. Flow cytometry was only used for cell collection in this study. FlowJo 10.8.2 (Mac Only) was used to plot FACS gating strategy.
Cell population abundance	Cells post-sort were immediately loaded for single cell RNAseq. Based on the scRNAseq results, we found that true McSCs (based on detection of known McSC markers) that are labeled by GFP composed 95.2-97.6% of total sequenced cells.
Gating strategy	Living cells were selected by forward scatter, side scatter, doublets discrimination and by DAPI dye exclusion. Melanocytes were selected based on the expression of GFP reporter.

☒ Tick this box to confirm that a figure exemplifying the gating strategy is provided in the Supplementary Information.
